# Supplementary material for: Combination of AID2 and BromoTag expands the utility of degron-based protein knockdowns
Source: EMBO Rep. 2024 Aug 23;25(9):4062–77. doi: 10.1038/s44319-024-00224-4 (PMC11387839; doi:10.1038/s44319-024-00224-4)
Supplement: Supplementary file 1 — Appendix [file 44319_2024_224_MOESM1_ESM.pdf]

Appendix

Table of content

Appendix Figure S1. Schematic illustration showing the AID2, dTAG and BromoTag systems. (page 2)

Appendix Figure S2. Comparison of the AID2, dTAG and BromoTag systems using a GFP reporter. (page 3–4)

Appendix Figure S3. Re-expression of the reporter after depletion by the AID2, dTAG or BromoTag system. (page 5)

Appendix Figure S4. Related to Fig. 4. (page 6)

Appendix Figure S5. DNA damage foci formation in mAB-ORC1 and mAB-CDC6 cells. (page 7)

Appendix Figure S6. Double-degron with mAID and BromoTag enhances CDC6 depletion and confers profound defects in DNA replication. (pages 8–9)

Appendix Figure S7. The 2mAID tag confers lower the expression level of ORC1 and CDC6. (page 10)

Appendix Figure S8. Combinational depletion of ORC1 or CDC6 by siRNA and AID2-BromoTag enhances defects in S phase progression. (page 11)

Appendix Figure S9. The mAB-ORC1 mAB-CDC6 cells enter mitosis without DNA replication after ORC1 and CDC6 co-depletion. (page 12)

Appendix Reference (page 13)

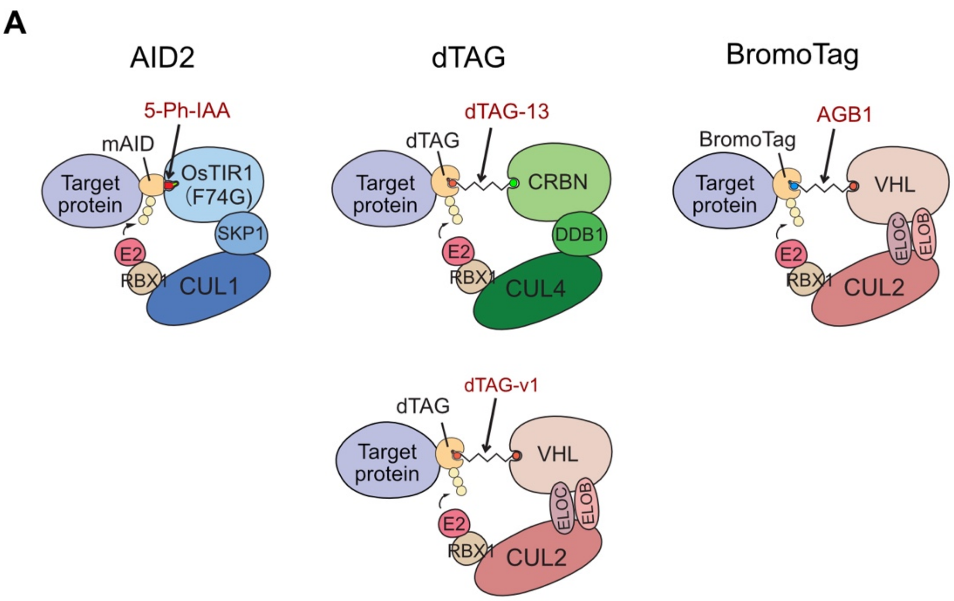

Degron	mini-AID (mAID) = IAA17(65–132aa)	dTAG = FKBP12(F36V)	BromoTag = BRD4-BD2(L387A)
Degron size	7.5 kDa	11.7 kDa	14.8 kDa
Ligand	5-Ph-IAA	dTAG-13, dTAG-v1	AGB1
E3 ligase	CRL1–OsTIR1(F74G)	CRL4–CRBN, CRL2–VHL	CRL2–VHL

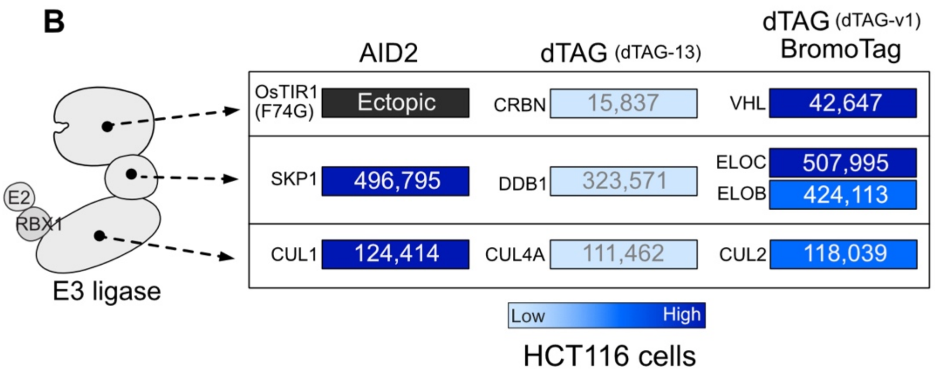

Appendix Figure S1

(**A**) Schematic illustration showing the AID2, dTAG and BromoTag systems. The table shows degron size, inducing ligand and the E3 ligase involved in target degradation. (**B**) The expression level of each E3 ligase component in HCT116 cells, originated from the paper by Bekker-Jensen et al (Bekker-Jensen *et al*, 2017).

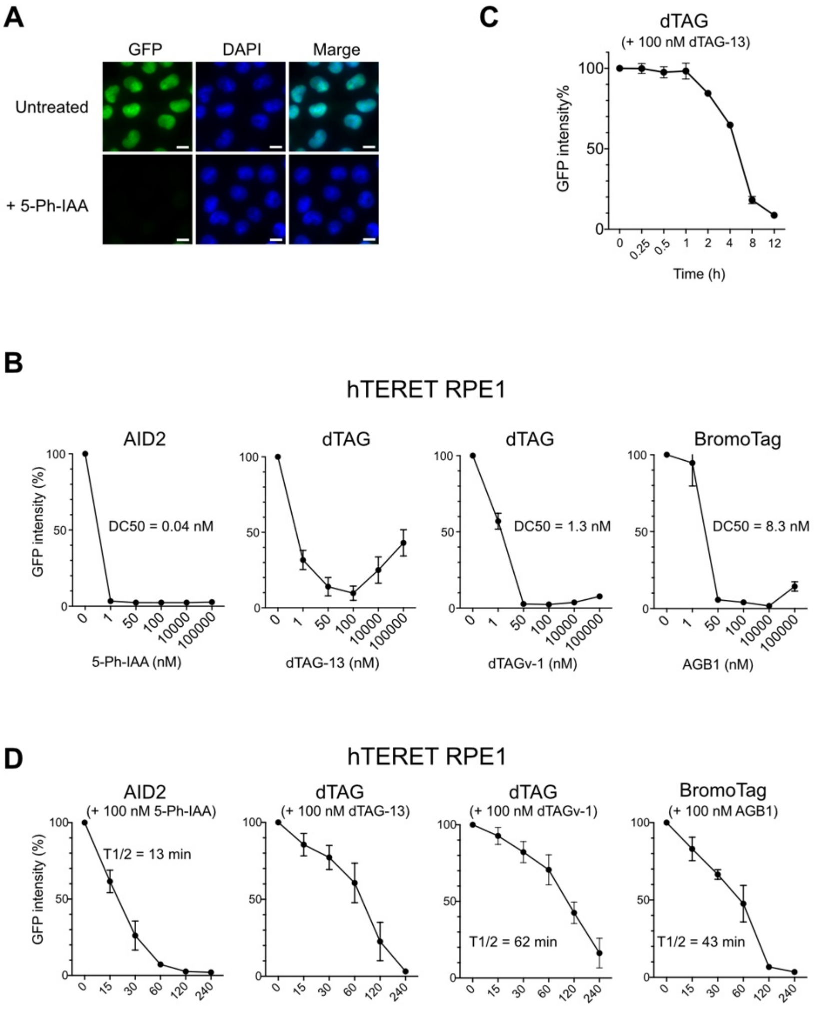

Appendix Figure S2

Comparison of the AID2, dTAG and BromoTag systems using a GFP reporter. (**A**) Representative fluorescent microscopic images of the HCT116 dTAG-BromoTag-mAID-EGFP-NLS reporter cells. The cells were treated with or without 1 µM 5-Ph-IAA for 2 h before fixation. The nuclei were stained with DAPI. Scale bars: 10 µm. (**B**) Dose-response of reporter depletion. The hTERT-RPE1 reporter cells were treated with the indicated concentrations of each ligand for 4 h. GFP intensity was analyzed taking the mock-treated cells as 100%. Data were presented as mean ± SD of three technical replicates (n=3). The DC50 values were calculated with the non-linear regression model on Graphpad Prism 8. (**C**) Time-course depletion of the reporter. The HCT116 GFP reporter cells were treated with 100 nM dTAG-13 as in **Fig. 2C**. Time-course samples were taken up to 12 h. (**D**) Time-course depletion of the reporter in hTERT-RPE1 cells. Cells were treated with 100 nM of the indicated ligand. Samples were taken at the indicated time point. GFP intensity was analyzed taking the mock-treated cells as 100%. Data were presented as mean ± SD of three

technical replicates (n=3). The T1/2 was calculated with the non-linear regression model on Graphpad Prism 8.

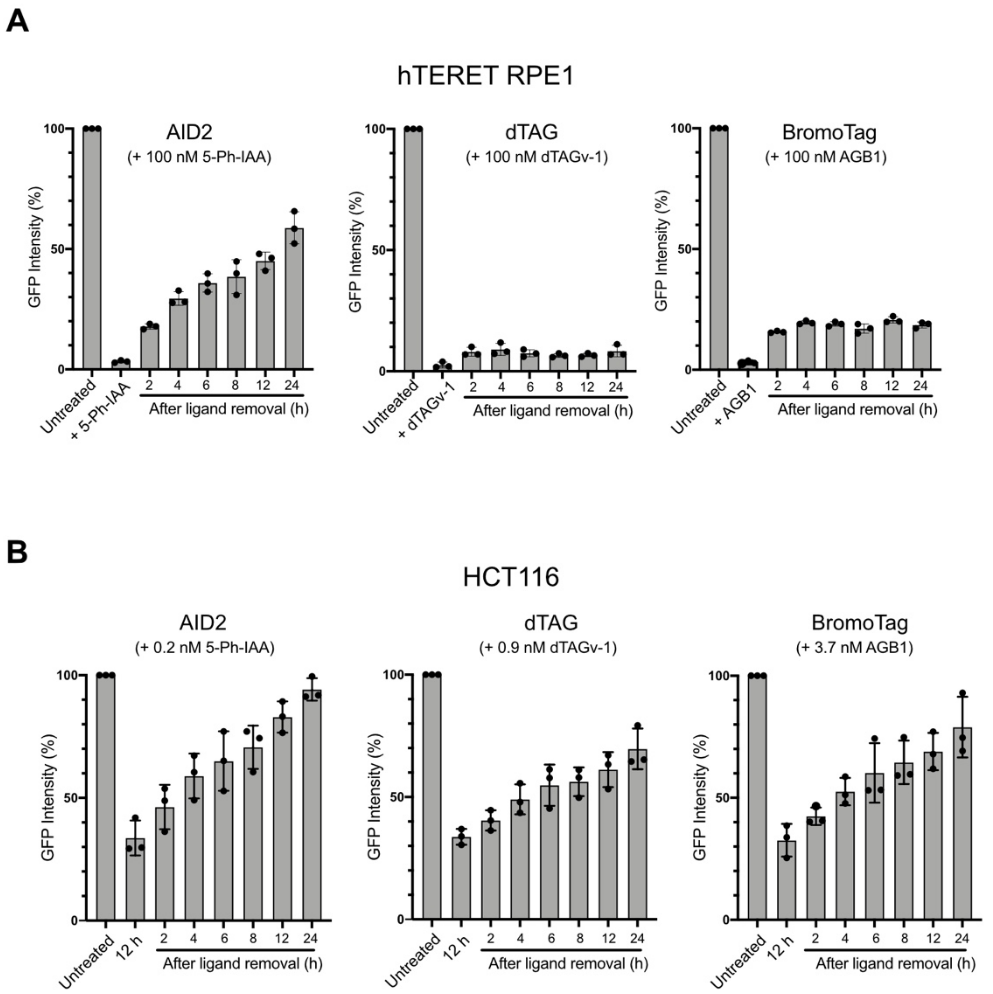

Appendix Figure S3

(**A**) Re-expression of the reporter after depletion by the AID2, dTAG or BromoTag system in hTERT REP1 cells. The reporter cells were treated with 1nM 5-Ph-IAA, 100 nM dTAGv-1 or 100 nM AGB1 for 4 h before medium change. Samples were taken at the indicated time points, and the GFP intensity was analyzed by taking the mock-treated cells as 100%. Data were presented as mean ± SD. Each dot represents a technical replicate (n=3). (**B**) The HCT116 GFP reporter cells were treated with 0.5 nM 5-Ph-IAA, 0.9 nM dTAGv-1 or 3.7 nM AGB1 for 12 h to lower the reporter level down to approximately 30%. Subsequently, the cells were washed, and re-expression of the reporter was monitored up to 24 h. Data were presented as mean ± SD. Each dot represents a technical replicate (n=3).

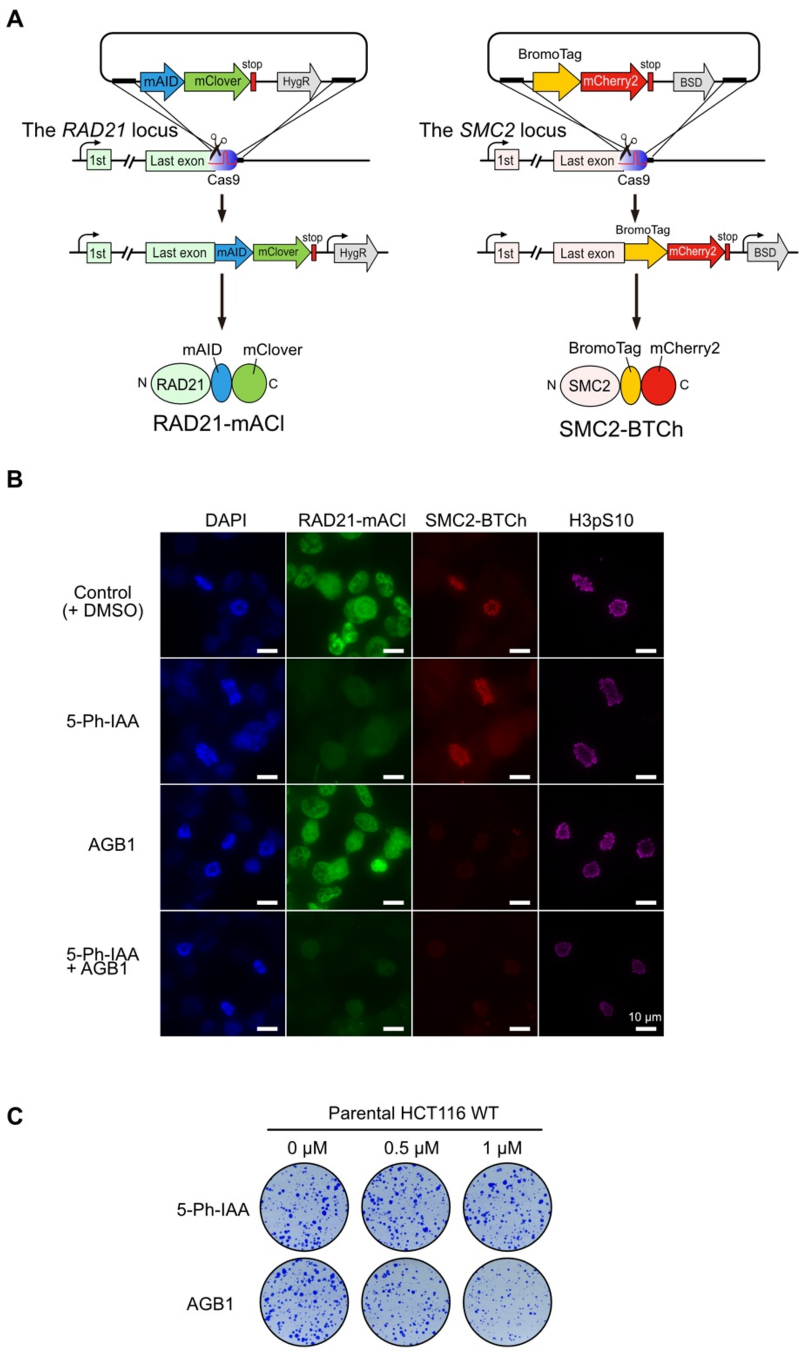

Appendix Figure S4

Related to Fig. 4. (**A**) Illustration showing the construction cells expressing RAD21-mAID-mClover (RAD21-mAC) and SMC2-BromoTag-mCherry2 (SMC2-BCh). (**B**) Fluorescent microscopic images of the RAD21-mAC/SMC2-BTCh cell line in the presence and absence of 1μM 5-Ph-IAA and/or 0.5 μM AGB1 for 4 h. Mitotic cells were stained with anti p-Histone H3 (Ser10) antibody. Ten slices taken every 0.5 μm were stacked. Scale bar: 10 μm. (**C**) Testing side effects of 5-Ph-IAA and AGB1 by colony formation. The parental HCT116 WT cells were cultured with the indicated dose of 5-Ph-IAA or AGB1 for 7 days. Colonies were stained with crystal violet.

A

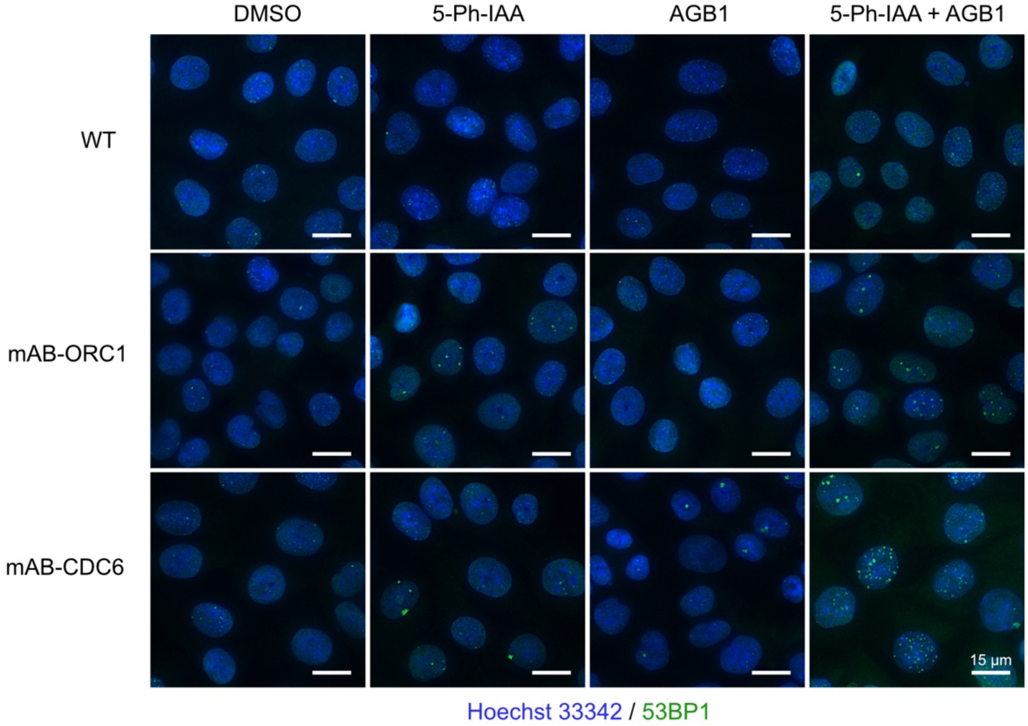

B

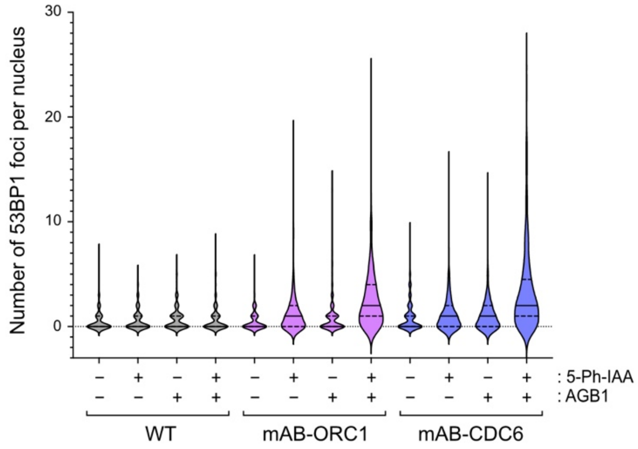

Appendix Figure S5

DNA damage foci formation in mAB-ORC1 and mAB-CDC6 cells. (**A**) Representative 53BP1 immunofluorescence images after treating the indicated cells with 1 μM 5-Ph-IAA, 0.5 μM AGB1 or both for 43 h. 53BP1 and DNA are shown in green and blue, respectively. (**B**) The number of 53BP1 foci per nucleus was quantified and presented in the violin plot. The solid lines show the median and dashed lines show the quartiles. More than 250 nuclei were quantified in each condition.

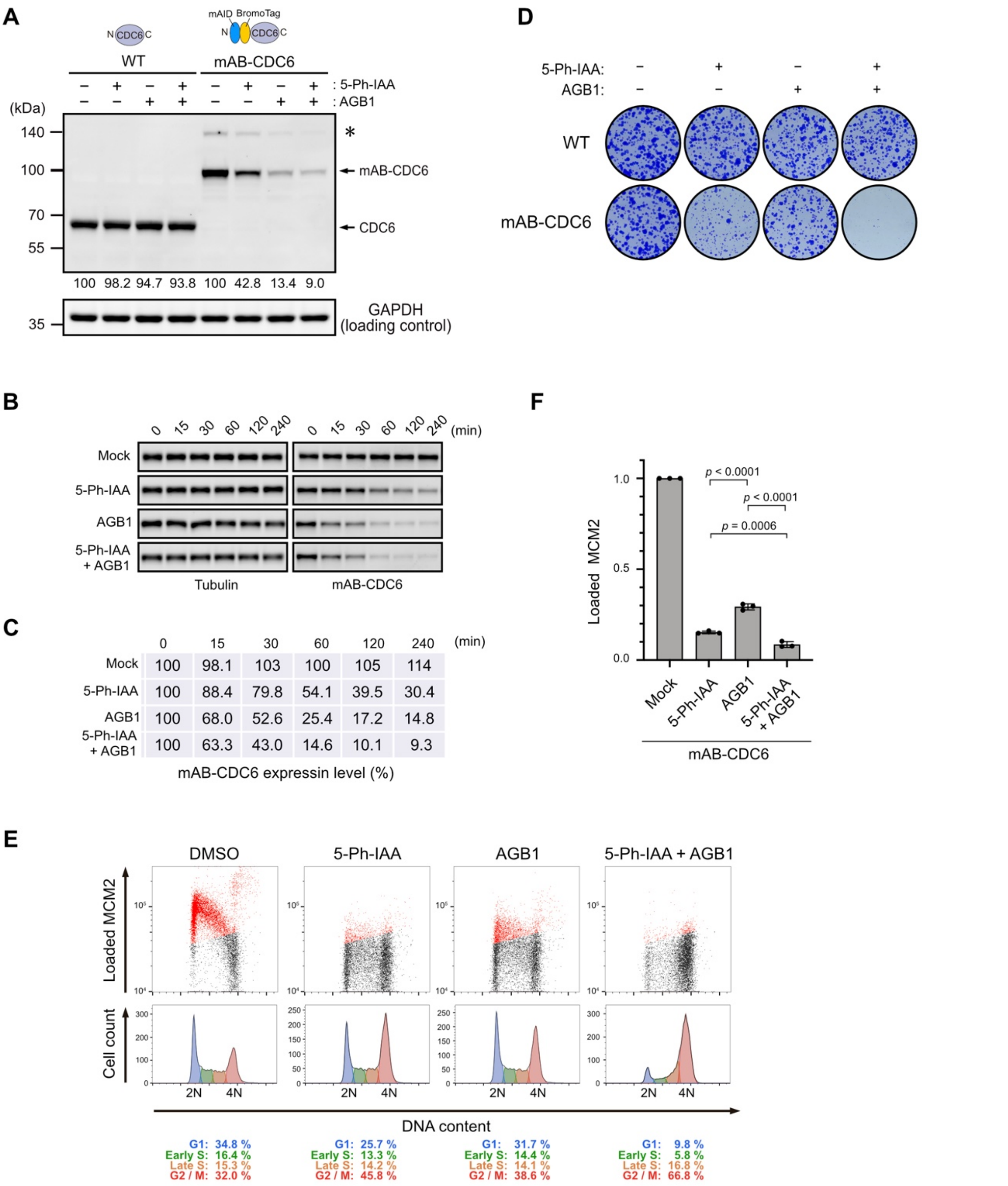

Appendix Figure S6

Double-degron with mAID and BromoTag enhances CDC6 depletion and confers profound defects in DNA replication. (**A**) The parental HCT116 wild-type (WT) and mAID-BromoTag-CDC6 (mAB-CDC6) cells were treated with 1 µM 5-Ph-IAA, 0.5 µM AGB1 or both for 2 h. Proteins were detected by anti-CDC6 and -GAPDH antibodies. Relative CDC6 levels taking the DMSO-treated control as 100% were shown under each blot. Each data was normalized with the corresponding tubulin loading control. The asterisk indicates the HygR-P2A-mAID-BromoTag-CDC6 protein before self-cleavage at the P2A site. (**B**) Depletion kinetics of mAB-CDC6 in cells treated with 1

μM 5-Ph-IAA and/or 0.5 μM AGB1. Samples were taken at the indicated time points. (**C**) The blot data in panel B were quantified taking the 0 min sample as 100%. Each data was normalized with the corresponding tubulin loading control. (**D**) Colony formation of the parental HCT116 WT and mAB-CDC6 cells. Cells were cultured in the presence or absence of 1 μM 5-Ph-IAA and/or 0.5 μM AGB1 for 7 days. Colonies were stained with crystal violet. (**E**) (Upper panels) Levels of chromatin-loaded MCM2 and DNA in mAB-CDC6 treated with 1 μM 5-Ph-IAA and/or 0.5 μM AGB1 for 24 h. The MCM2-positive cells are shown in red. (Lower panels) Cell count histogram to the same samples. The percentages of each cell cycle are shown below. (**F**) Levels of chromatin-loaded MCM2 in mAB-CDC6 cells treated with the indicated ligand. Data were presented as mean ± SD. Each dot represents a technical replicate (n=3). Cells were synchronized in M phase with 50 ng/mL nocodazole for 14 h and released into a fresh media containing ligand. Cells were treated with 1 μM 5-Ph-IAA and/or 0.5 μM AGB1 2 h prior to nocodazole release. Samples were taken at 4 h after release when cells were in G1. Statistical analysis was performed with Tukey’s multiple comparison test.

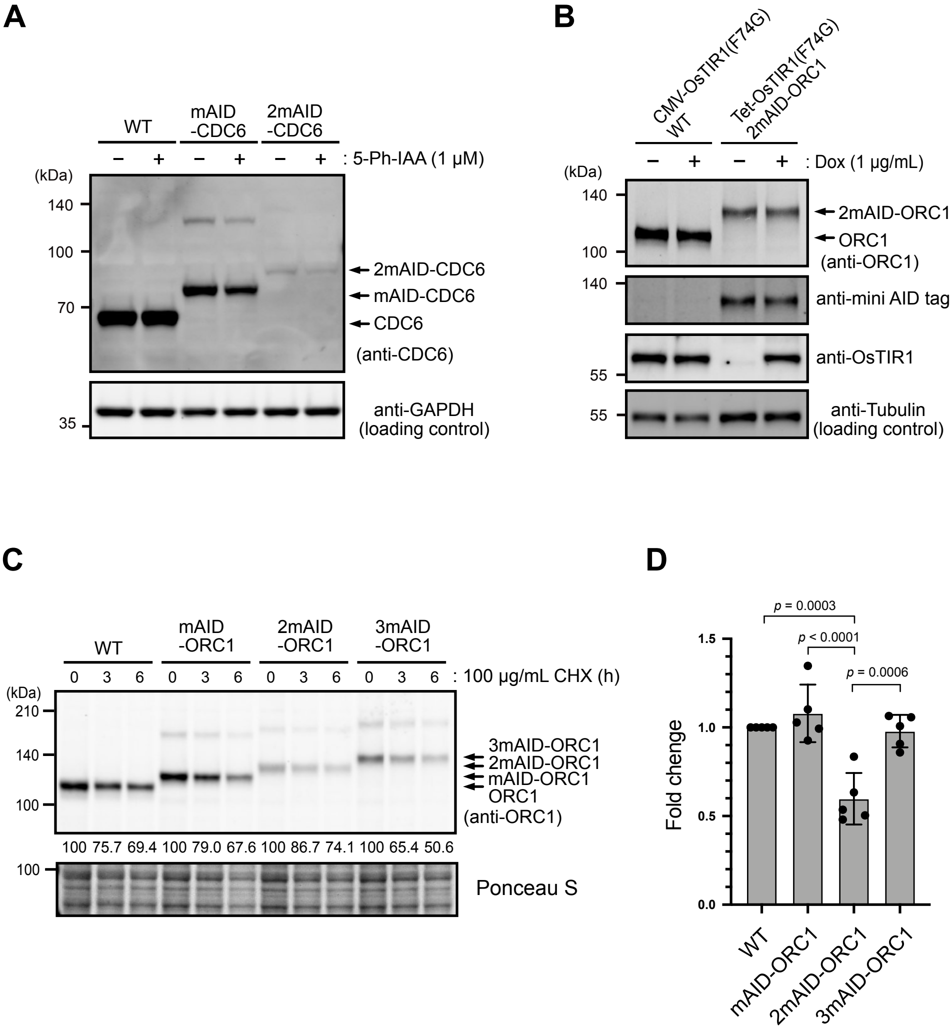

Appendix Figure S7

The 2mAID tag confers lower the expression level of ORC1 and CDC6. (**A**) CDC6 level in the indicated cells treated with or without 1μM 5-Ph-IAA for 2 h. Proteins were detected with the indicated antibodies. (**B**) We generated a cell line expressing 2mAID-ORC1 in the Tet-OsTIR1(F74G) background, in which OsTIR1(F74G) is induced by the addition of doxycycline (Dox). The parental HCT116 WT cells used in this study (CMV-OsTIR1 background) and the 2mAID-ORC1 (Tet-OsTIR1(F74G) background) cells were treated with 1 µg/mL doxycycline for 24 h. Proteins were detected by the indicated antibodies. (**C**) The half-life of ORC1 in the indicated cell lines. The cells treated with 100 µg/mL of cycloheximide (CHX) were cultured for 0, 3 and 6 h. The relative ORC1 levels adjusted by Ponceau S staining are shown under the ORC1 blot. (**D**) The ORC1 mRNA abundance in the indicated cell lines. Relative ORC1 mRNA expression levels were shown with WT set to 1.0. Data were presented as mean ± SD. Each dot represents a technical replicate (n=5). Statistical analysis was performed with Tukey's multiple comparison test.

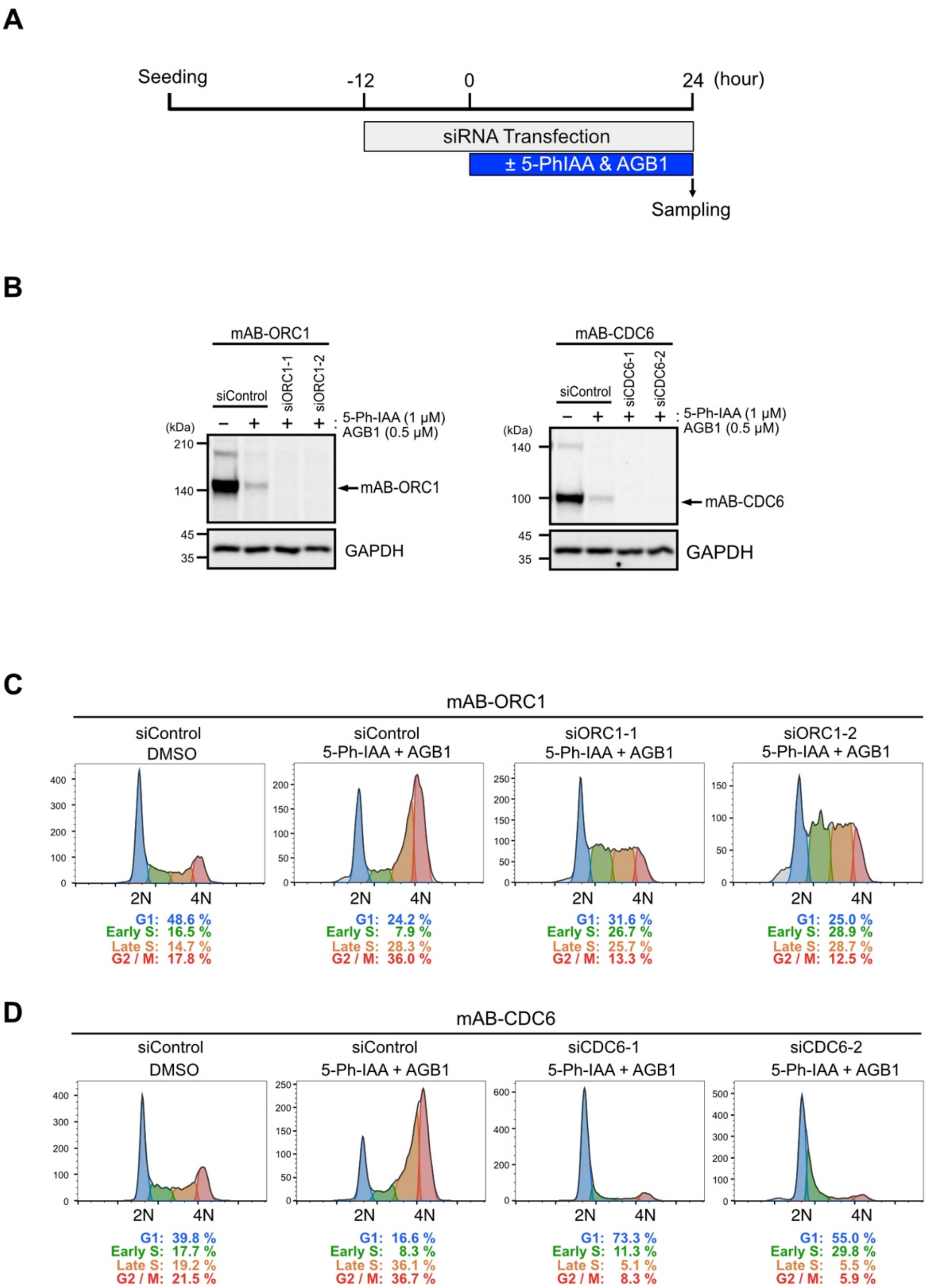

Appendix Figure S8

Combinational depletion of ORC1 or CDC6 by siRNA and AID2-BromoTag enhances defects in S phase progression. (**A**) Experimental scheme of depletion by combining siRNA and AID2-BromoTag. (**B**) The ORC1 and CDC6 levels after the combinational depletion. The indicated proteins were detected by anti-ORC1, anti-CDC6 and anti-GAPDH antibodies. (**C**)(**D**) The cell cycle profiles of the indicated cells were analyzed with flowcytometry. The percentages of each cell cycle are shown below.

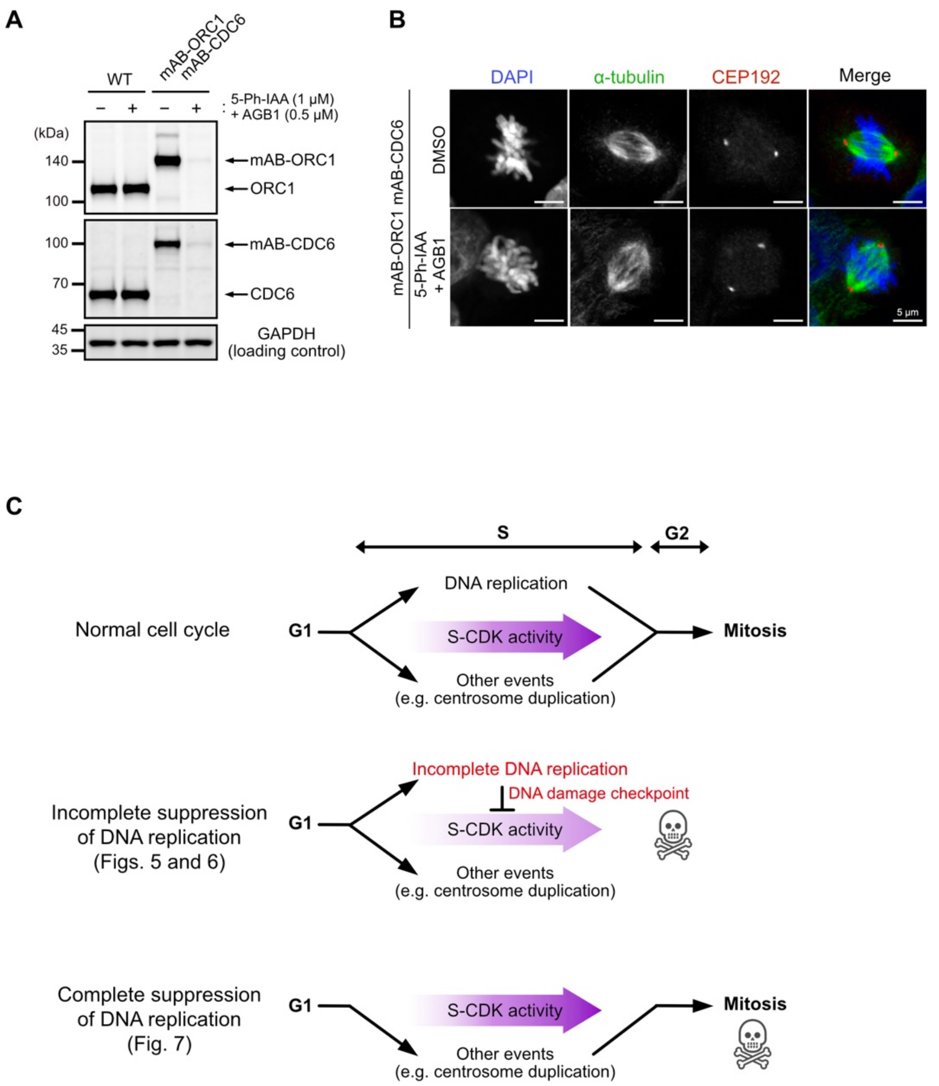

Appendix Figure S9

The mAB-ORC1 mAB-CDC6 cells enter mitosis without DNA replication after ORC1 and CDC6 co-depletion. (**A**) mAID-BromoTag was fused to the N-terminus of ORC1 and CDC6. The cells were treated with or without 1 µM 5-Ph-IAA and 0.5 µM AGB1 for 2 h before harvesting. Proteins were detected with anti-ORC1, -CDC6 and -GAPDH antibodies. (**B**) Confocal microscopic images of metaphase cells. DNA, mitotic spindle and centrosomes were stained by DAPI, anti-α-tubulin antibody and anti-CEP192 antibody, respectively. mAB-ORC1 mAB-CDC6 cells were cultured with or without 1 µM 5-Ph-IAA and 0.5 µM AGB1 for 24 h before fixation. (**C**) Illustration showing the relationship between DNA replication and the cell cycle control. Complete DNA suppression bypasses DNA replication resulting premature mitosis. Note that both incomplete and complete suppression of DNA replication leads to cell death. However, they were arrested at different cell cycle phases (late S/G2 and M, respectively).

Appendix Reference

Bekker-Jensen DB, Kelstrup CD, Batth TS, Larsen SC, Haldrup C, Bramsen JB, Sorensen KD, Hoyer S, Orntoft TF, Andersen CL *et al* (2017) An Optimized Shotgun Strategy for the Rapid Generation of Comprehensive Human Proteomes. *Cell Syst* 4: 587-599 e584
