# Supplementary figures and images for: Combination of AID2 and BromoTag expands the utility of degron-based protein knockdowns
Source: EMBO Rep. 2024 Aug 23;25(9):4062–77. doi: 10.1038/s44319-024-00224-4 (PMC11387839; doi:10.1038/s44319-024-00224-4)

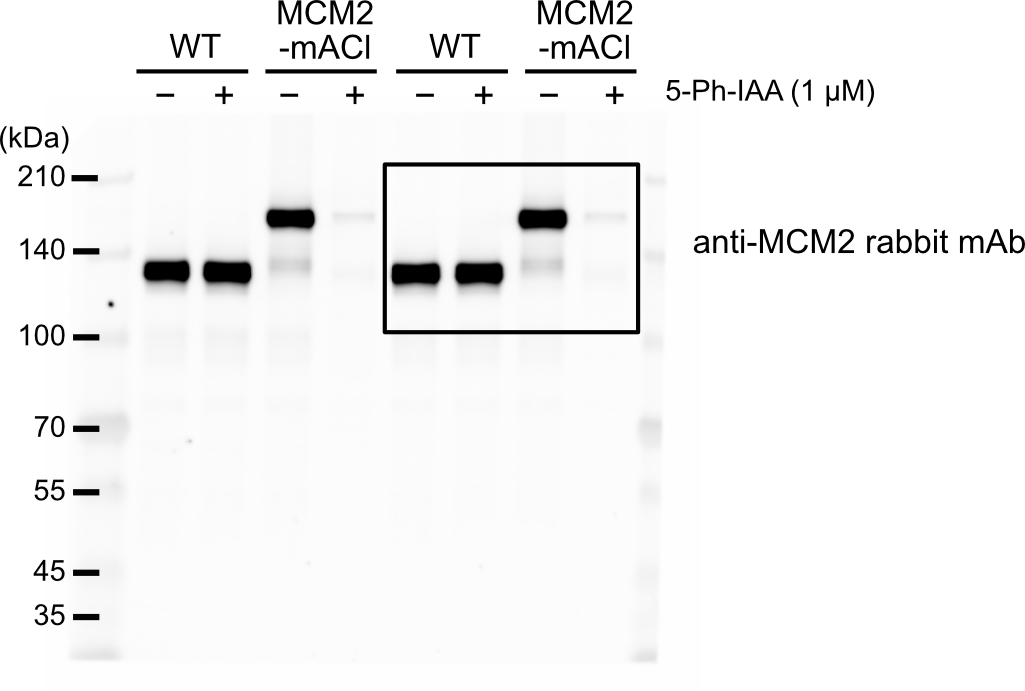

Supplement: Supplementary file 6 — Source data Fig. 1 [file 44319_2024_224_MOESM6_ESM.zip › Figure 1/1B/Upper_Western_MCM2.tiff]

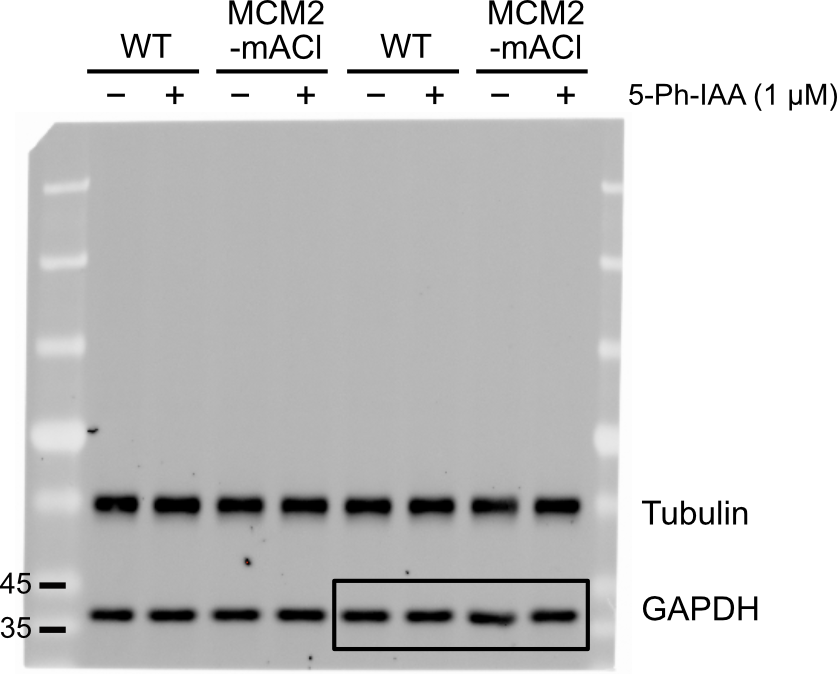

Supplement: Supplementary file 6 — Source data Fig. 1 [file 44319_2024_224_MOESM6_ESM.zip › Figure 1/1B/Upper_Western_GAPDH.tiff]

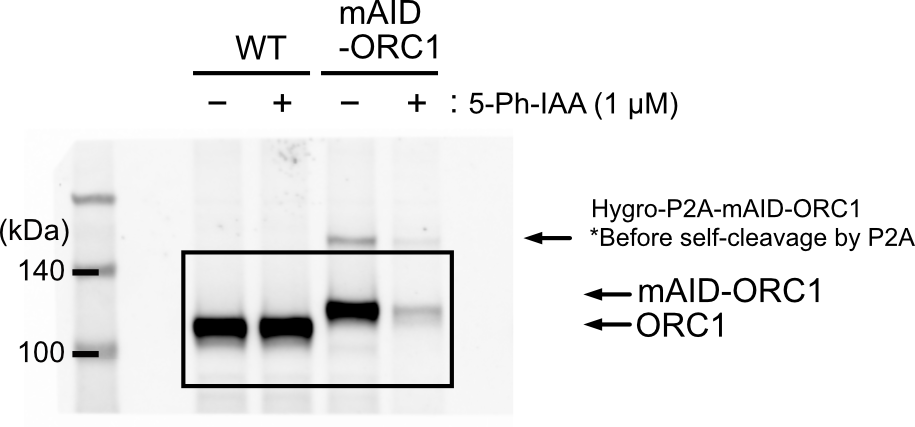

Supplement: Supplementary file 6 — Source data Fig. 1 [file 44319_2024_224_MOESM6_ESM.zip › Figure 1/1B/Lower_Western_ORC1.tiff]

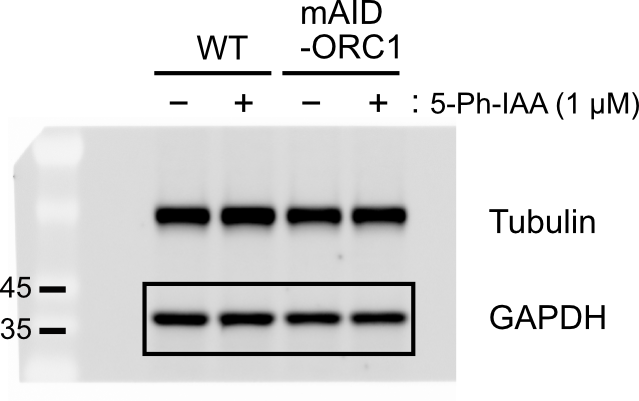

Supplement: Supplementary file 6 — Source data Fig. 1 [file 44319_2024_224_MOESM6_ESM.zip › Figure 1/1B/Lower_Western_GAPDH.tiff]

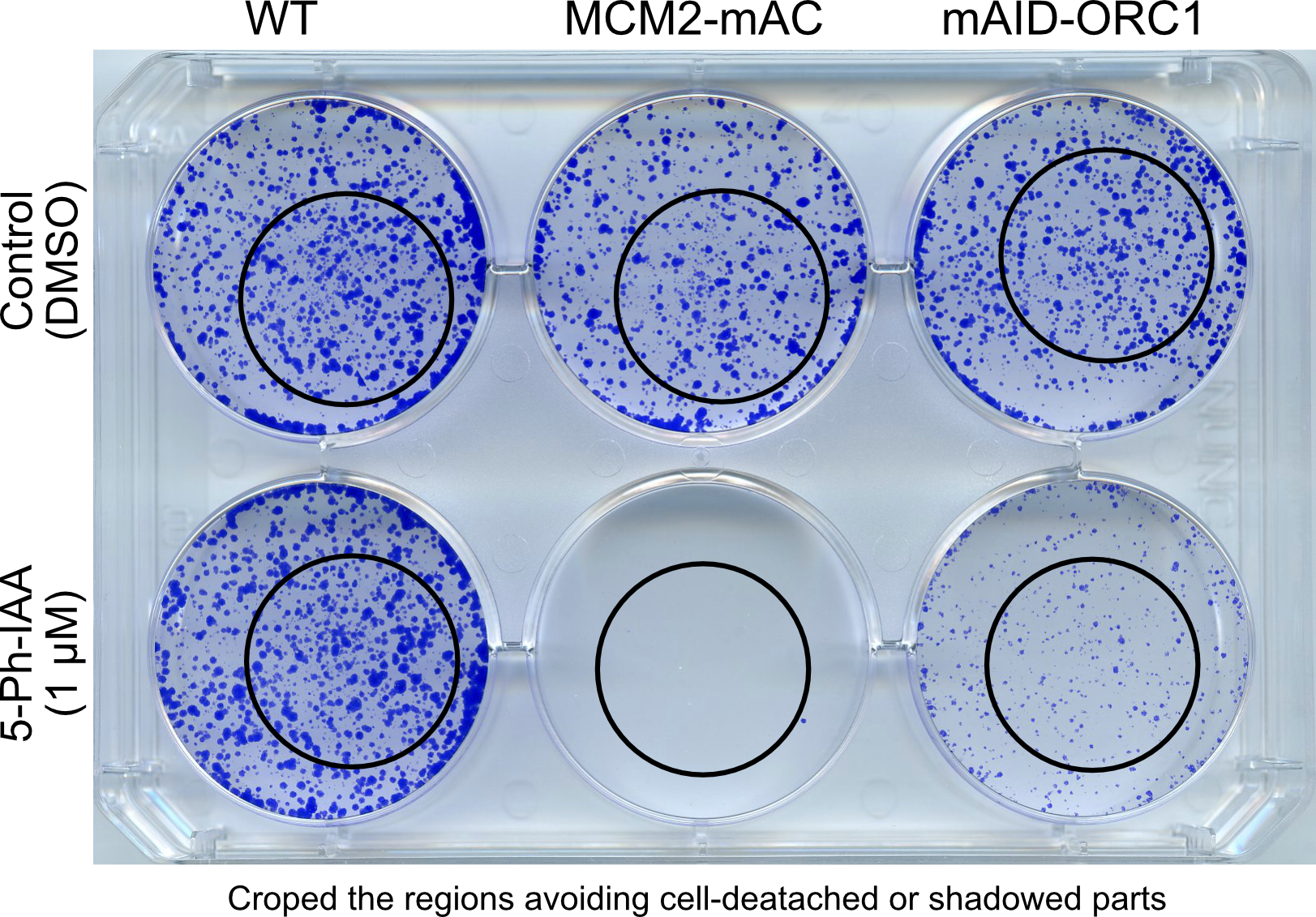

Supplement: Supplementary file 6 — Source data Fig. 1 [file 44319_2024_224_MOESM6_ESM.zip › Figure 1/1C/Colony formation assay_1C.tiff]

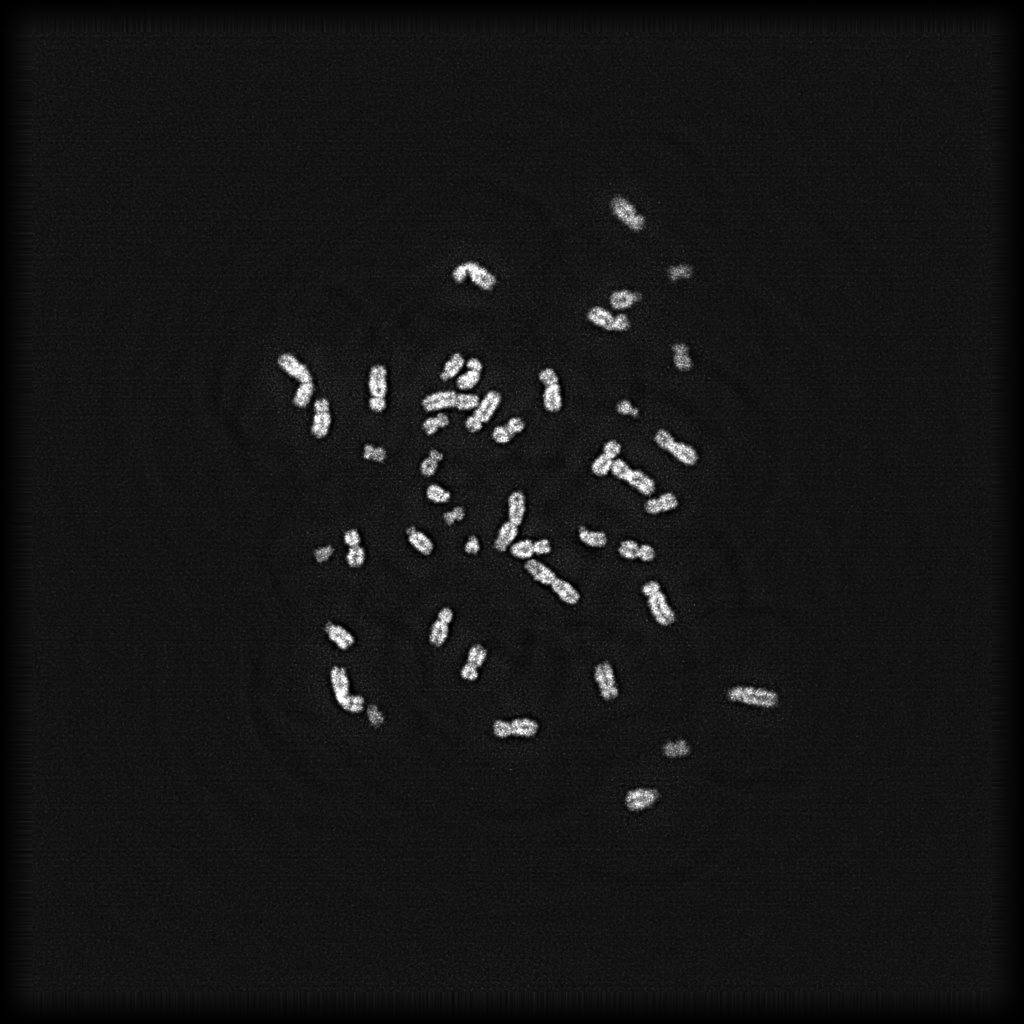

Supplement: Supplementary file 8 — Source data Fig. 3 [file 44319_2024_224_MOESM8_ESM.zip › Figure 3/3C/Spreads_deconvoluted/DMSO_decon.tif]

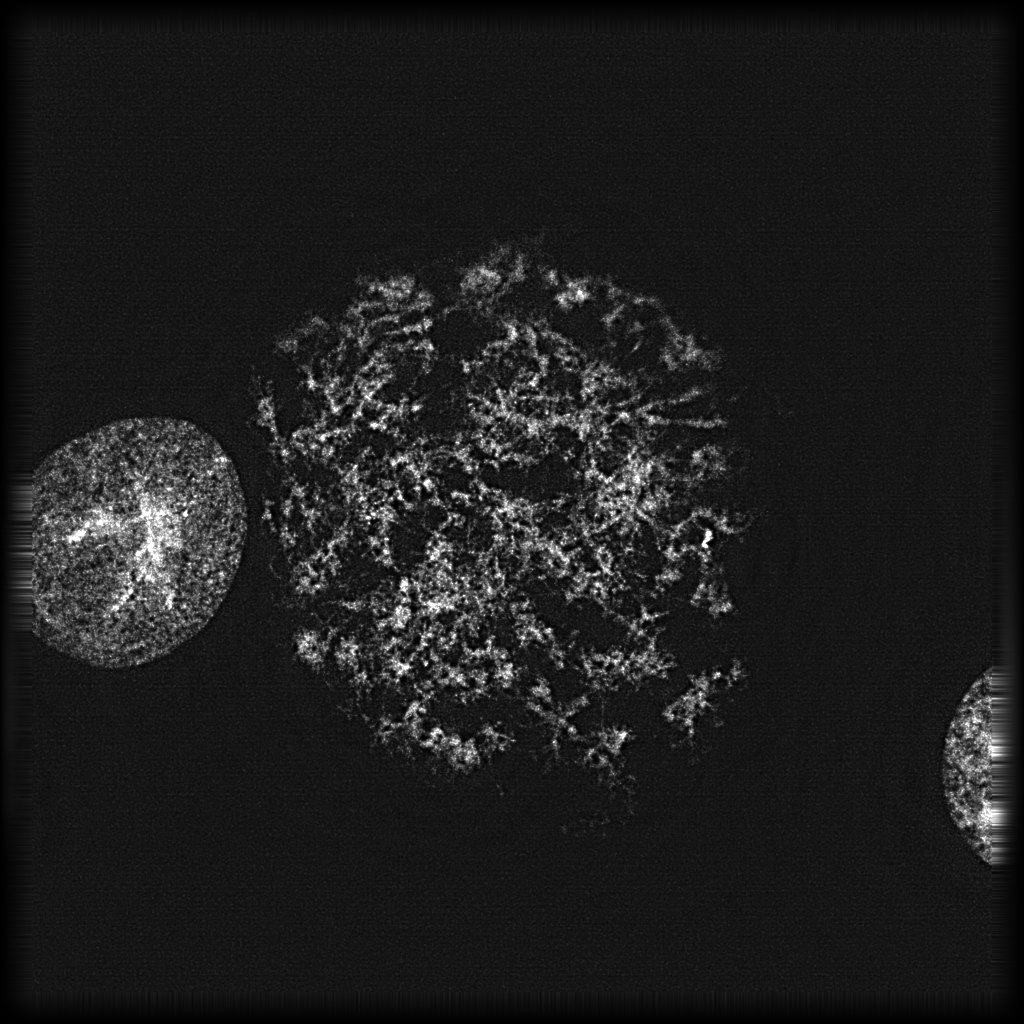

Supplement: Supplementary file 8 — Source data Fig. 3 [file 44319_2024_224_MOESM8_ESM.zip › Figure 3/3C/Spreads_deconvoluted/AGB1_decon.tif]

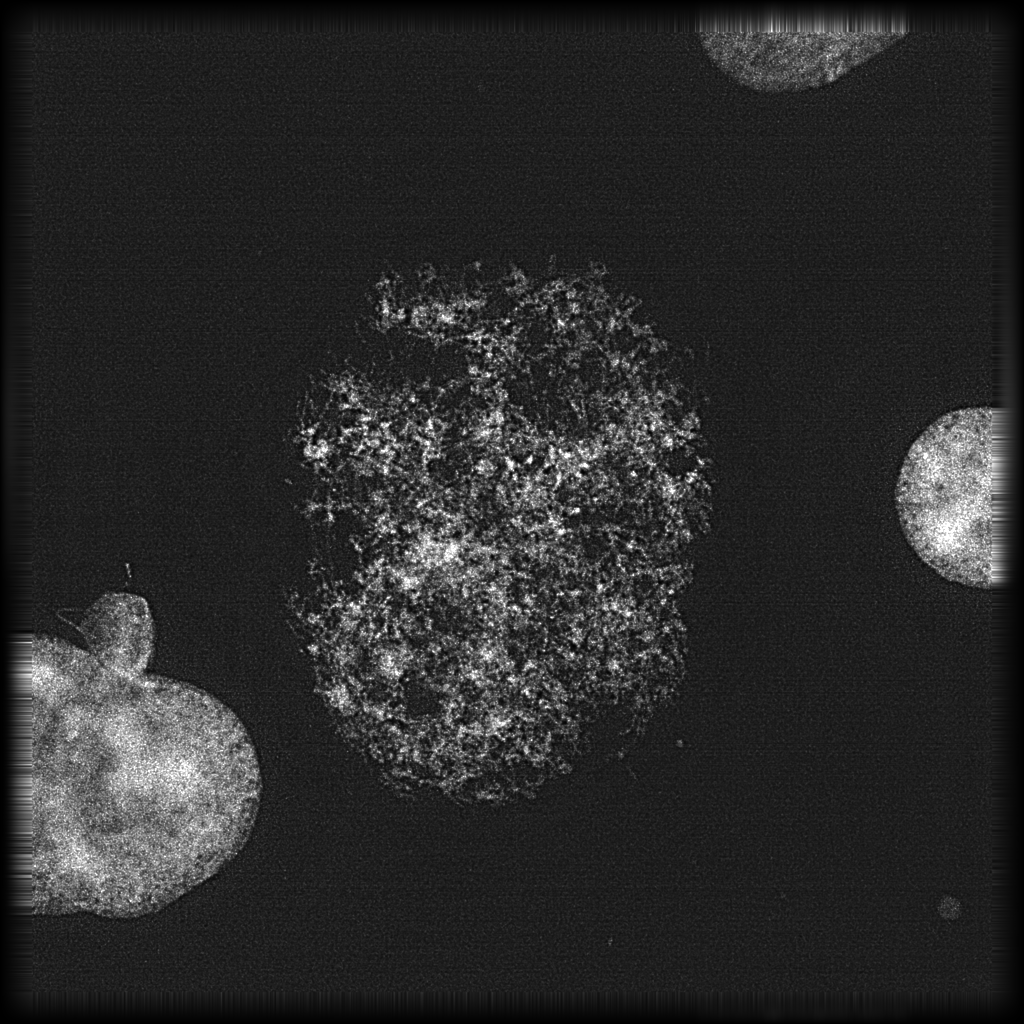

Supplement: Supplementary file 8 — Source data Fig. 3 [file 44319_2024_224_MOESM8_ESM.zip › Figure 3/3C/Spreads_deconvoluted/5-Ph-IAA+AGB1_decon.tif]

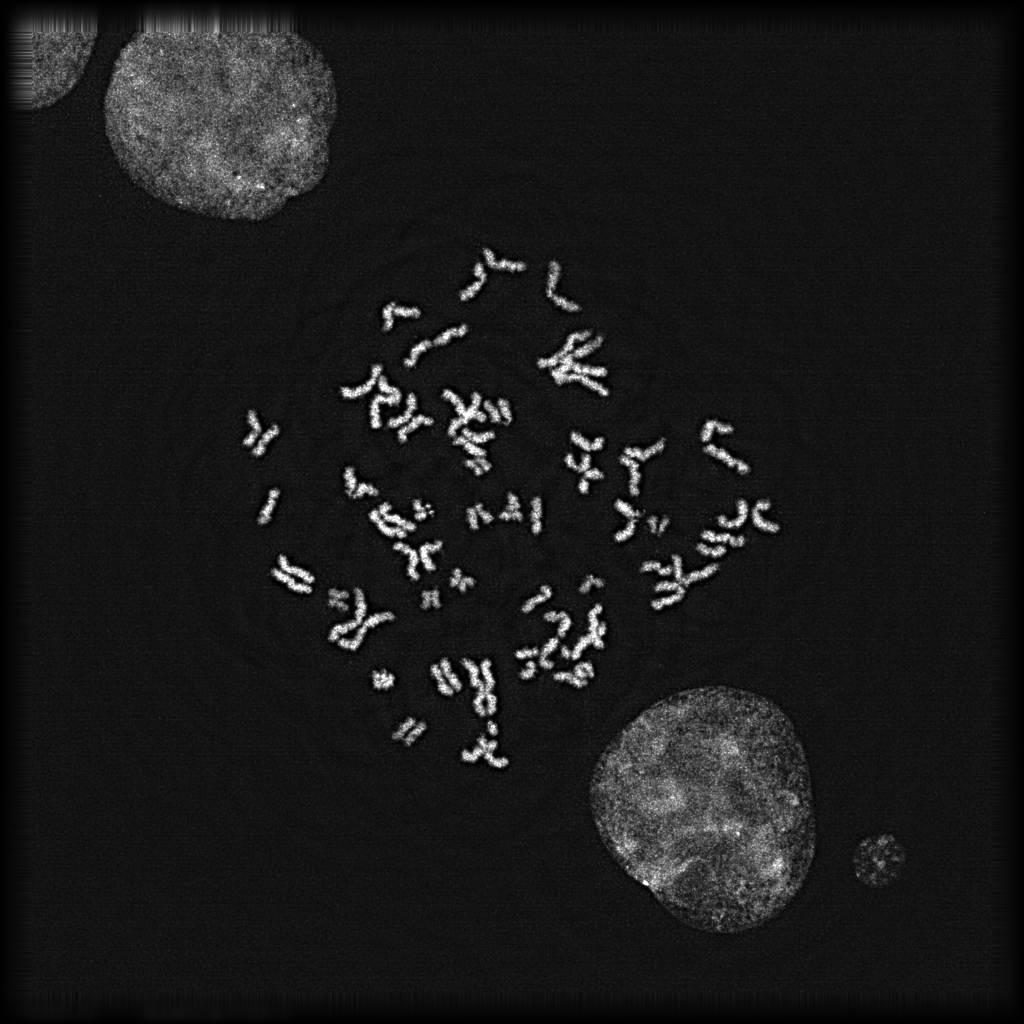

Supplement: Supplementary file 8 — Source data Fig. 3 [file 44319_2024_224_MOESM8_ESM.zip › Figure 3/3C/Spreads_deconvoluted/5-Ph-IAA_decon.tif]

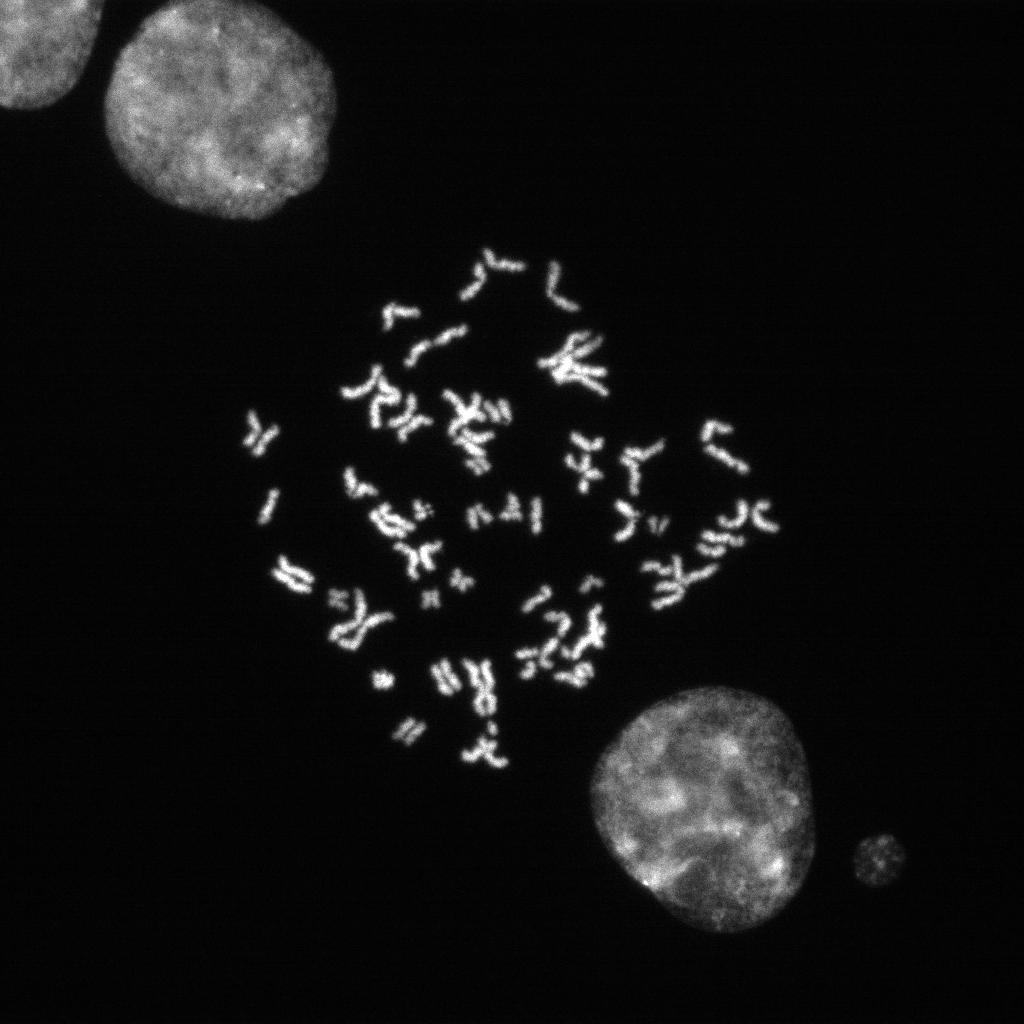

Supplement: Supplementary file 8 — Source data Fig. 3 [file 44319_2024_224_MOESM8_ESM.zip › Figure 3/3C/Spreads_raw data/5-Ph-IAA.tif]

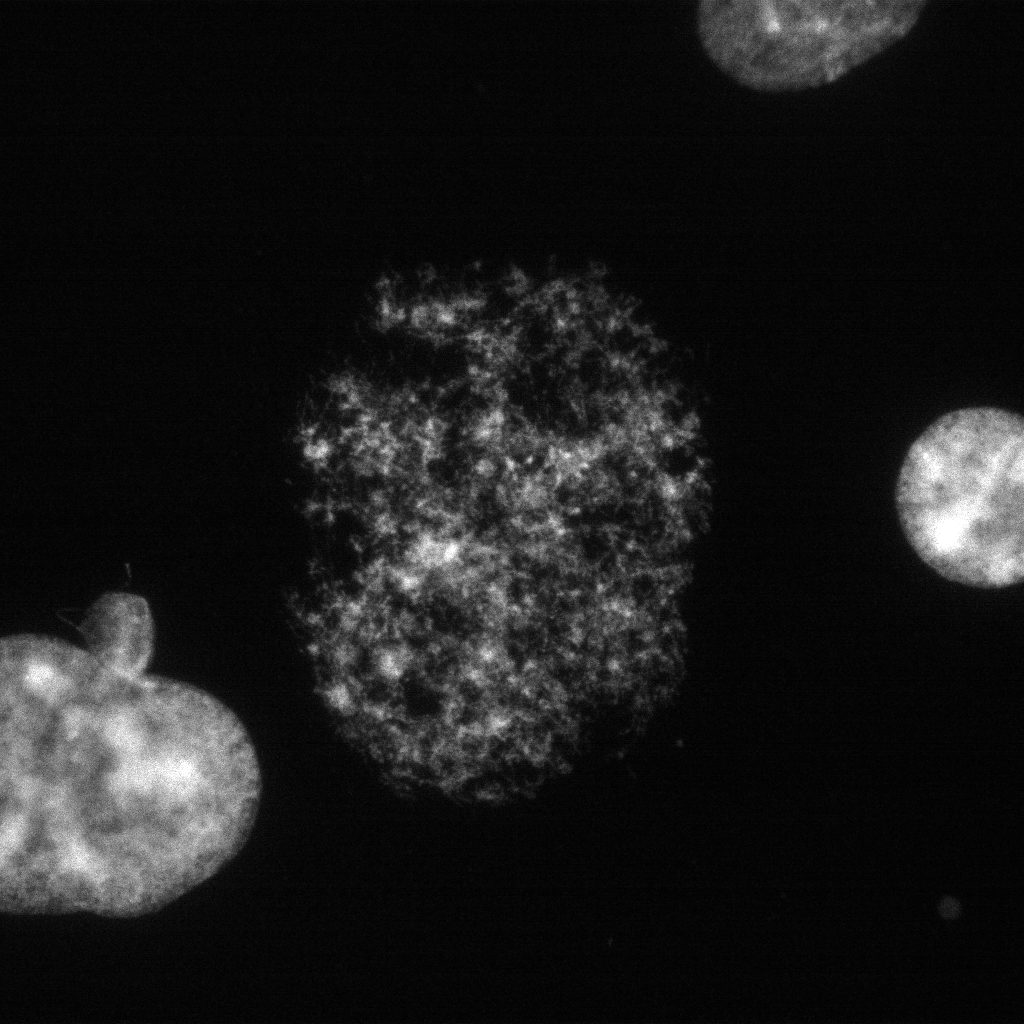

Supplement: Supplementary file 8 — Source data Fig. 3 [file 44319_2024_224_MOESM8_ESM.zip › Figure 3/3C/Spreads_raw data/5-Ph-IAA+AGB1.tif]

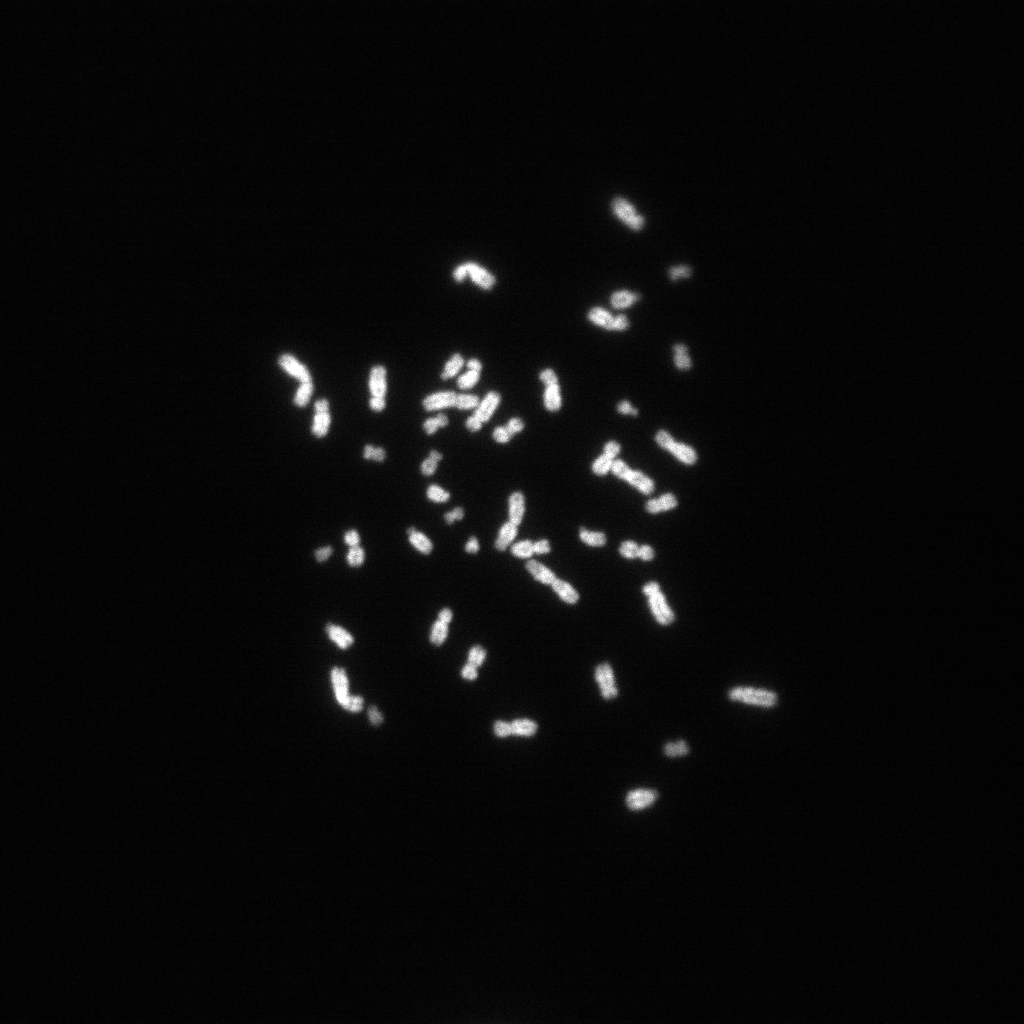

Supplement: Supplementary file 8 — Source data Fig. 3 [file 44319_2024_224_MOESM8_ESM.zip › Figure 3/3C/Spreads_raw data/DMSO.tif]

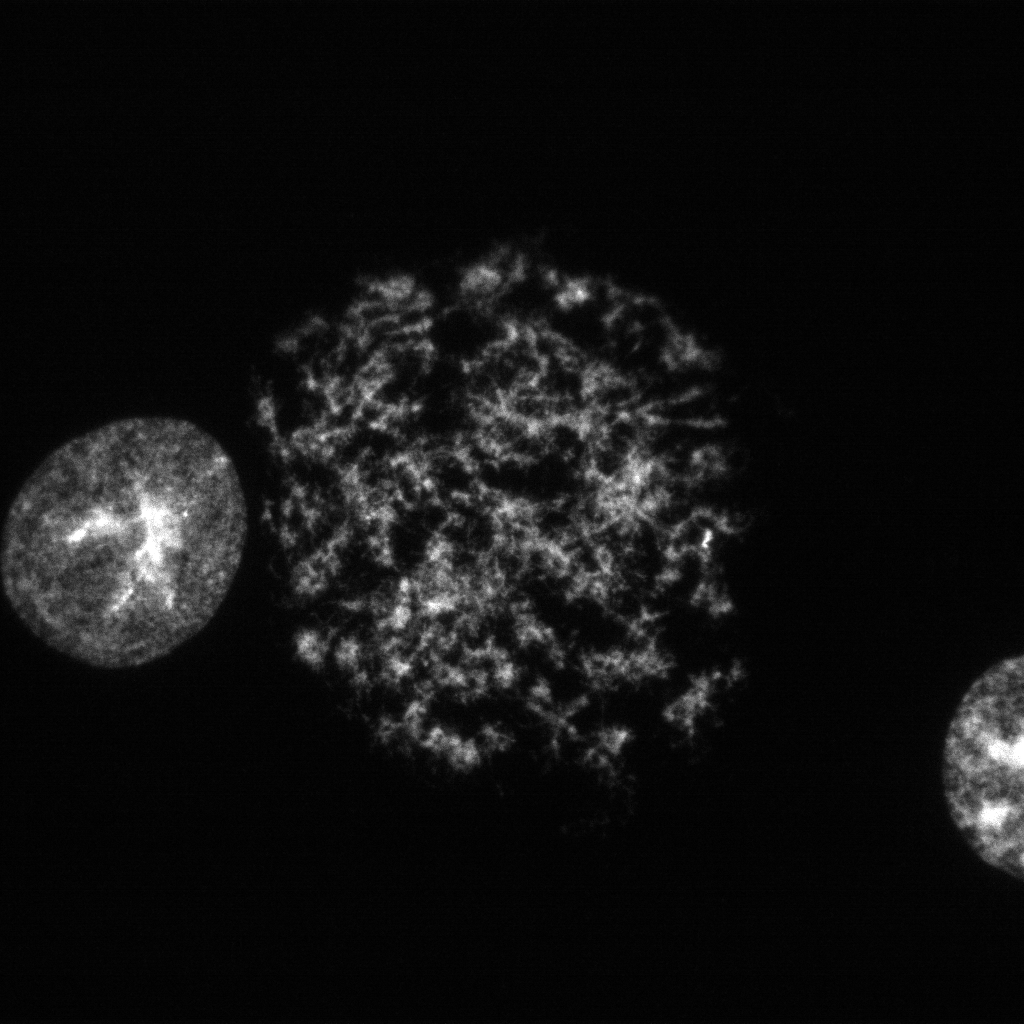

Supplement: Supplementary file 8 — Source data Fig. 3 [file 44319_2024_224_MOESM8_ESM.zip › Figure 3/3C/Spreads_raw data/AGB1.tif]

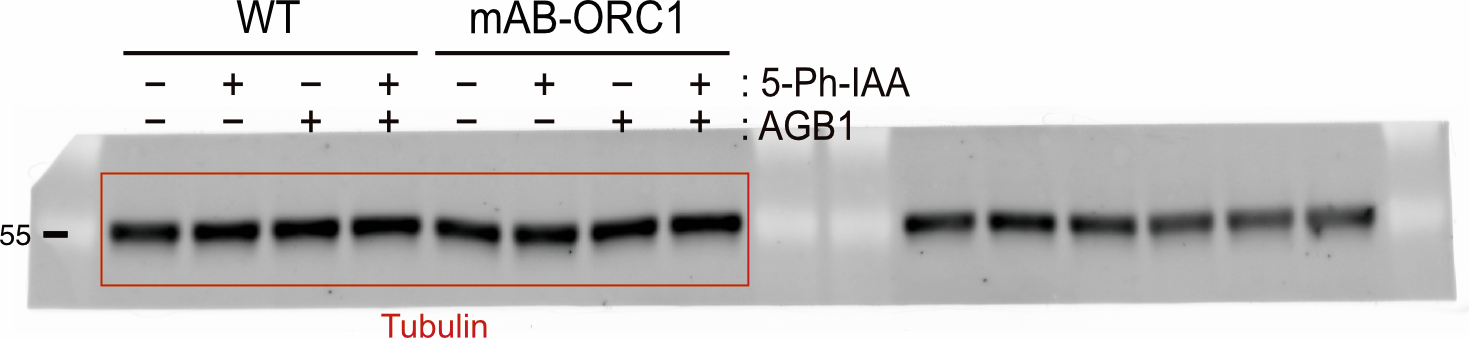

Supplement: Supplementary file 10 — Source data Fig. 5 [file 44319_2024_224_MOESM10_ESM.zip › Figure 5/5A/5A_Tubulin_WB.tiff]

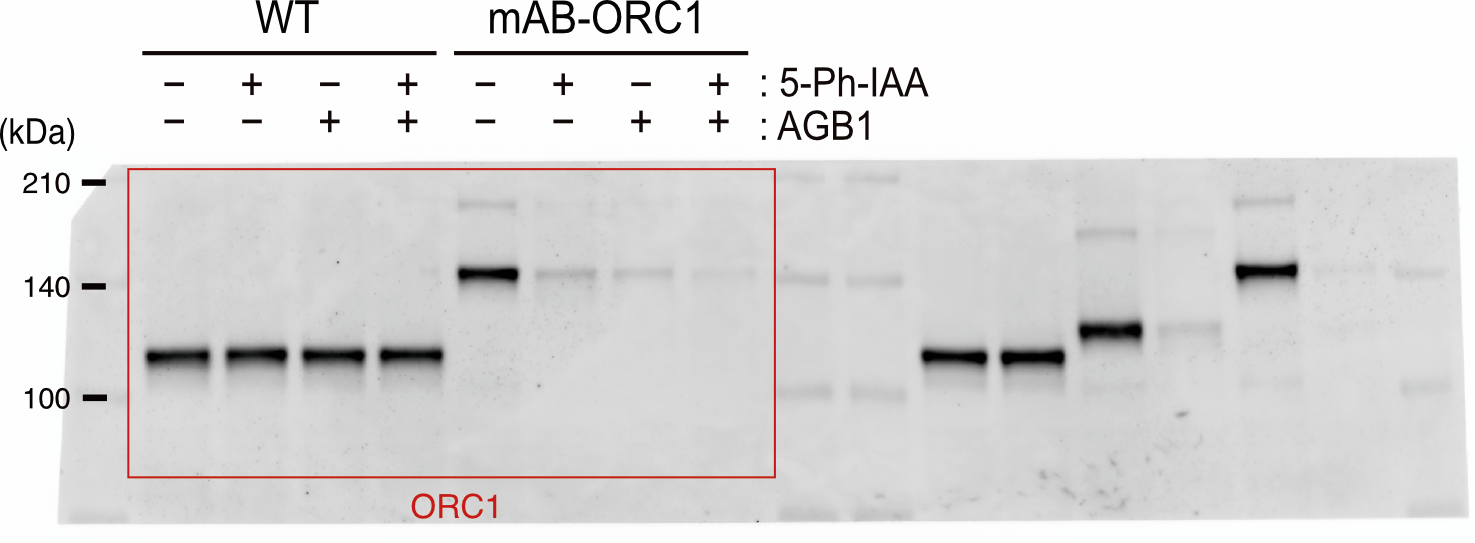

Supplement: Supplementary file 10 — Source data Fig. 5 [file 44319_2024_224_MOESM10_ESM.zip › Figure 5/5A/5A_ORC1_WB.tiff]

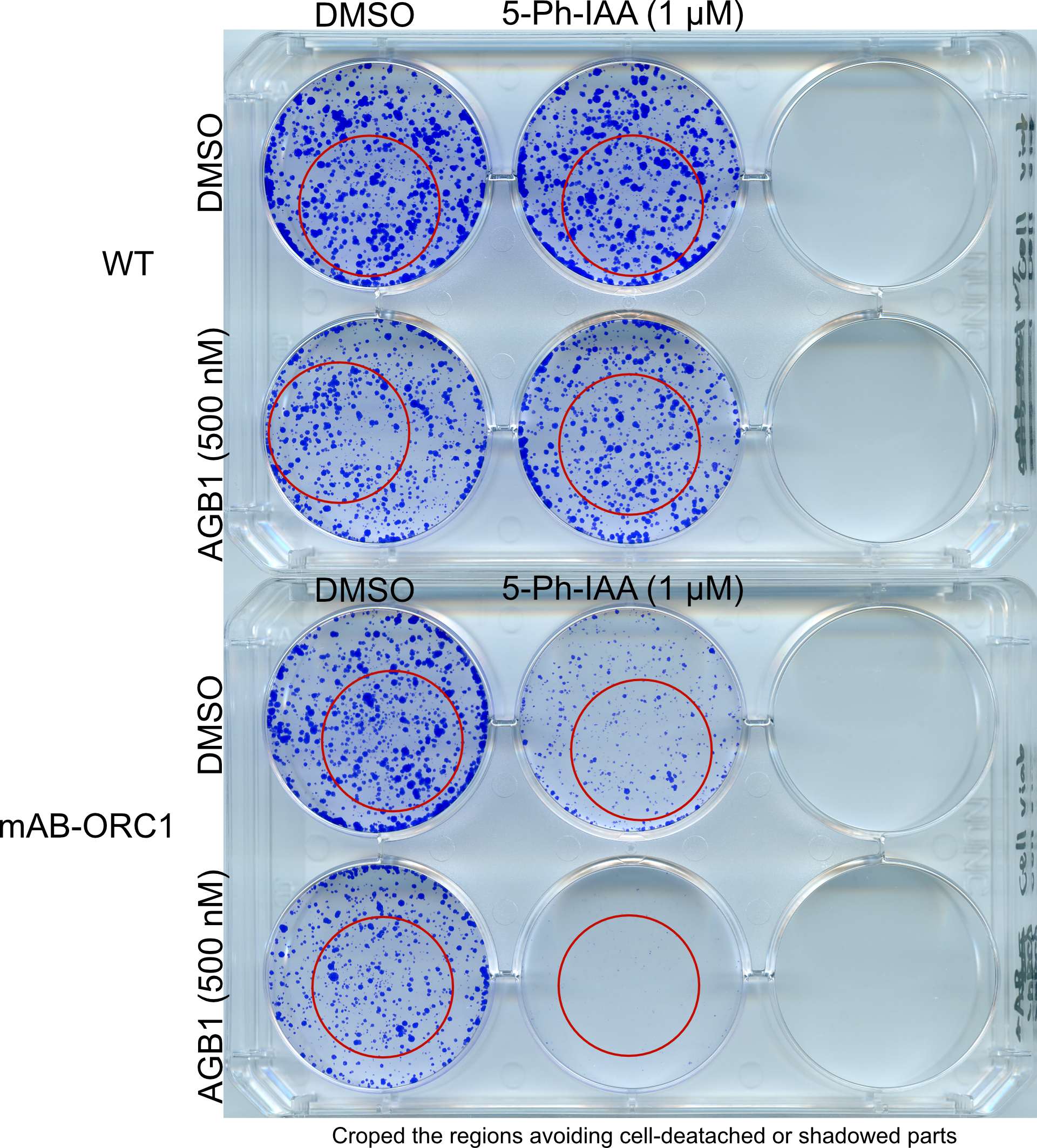

Supplement: Supplementary file 10 — Source data Fig. 5 [file 44319_2024_224_MOESM10_ESM.zip › Figure 5/5D/5D_Colony formation assay.tiff]

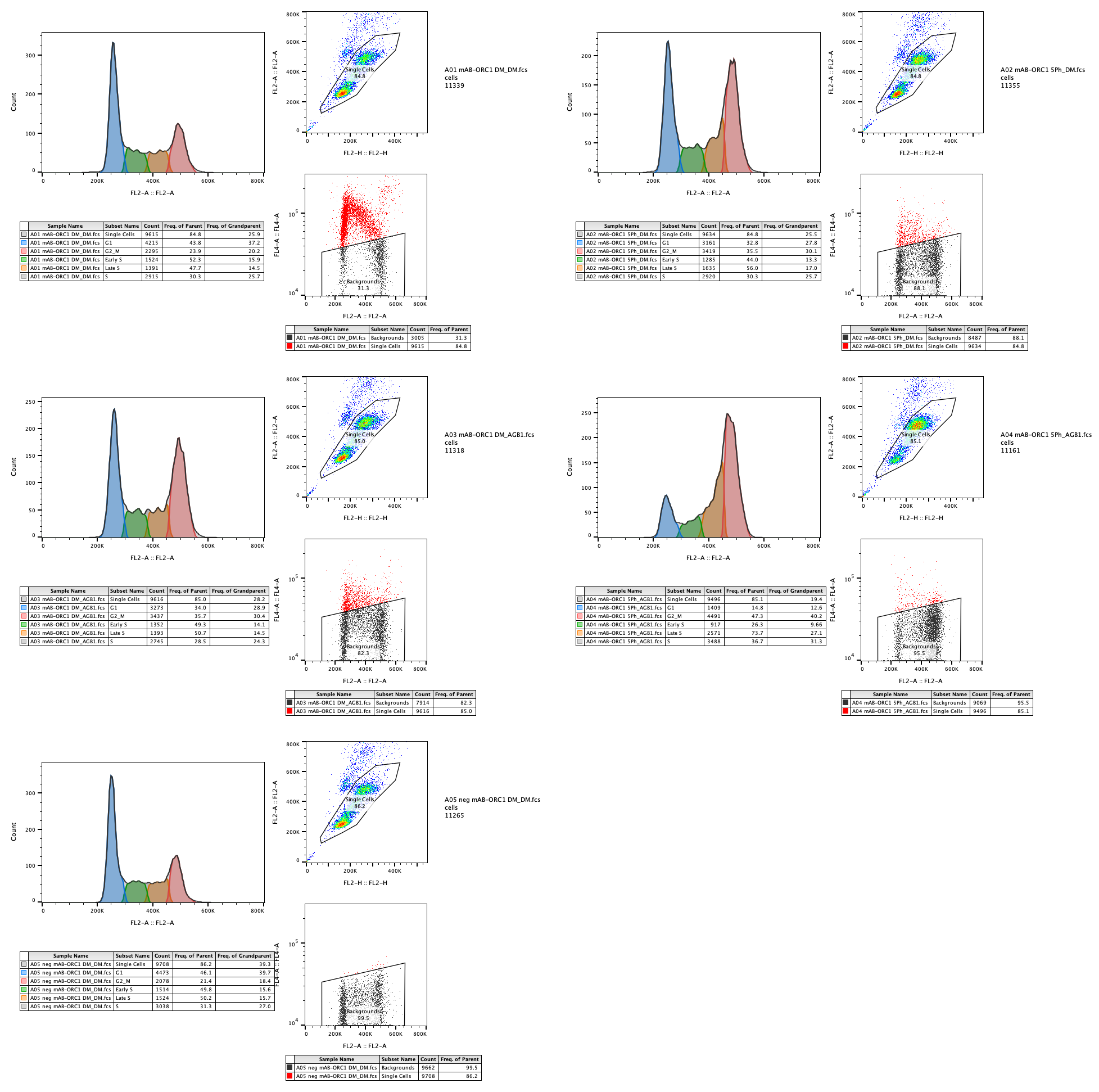

Supplement: Supplementary file 10 — Source data Fig. 5 [file 44319_2024_224_MOESM10_ESM.zip › Figure 5/5E/5E_Flowcyto.tiff]

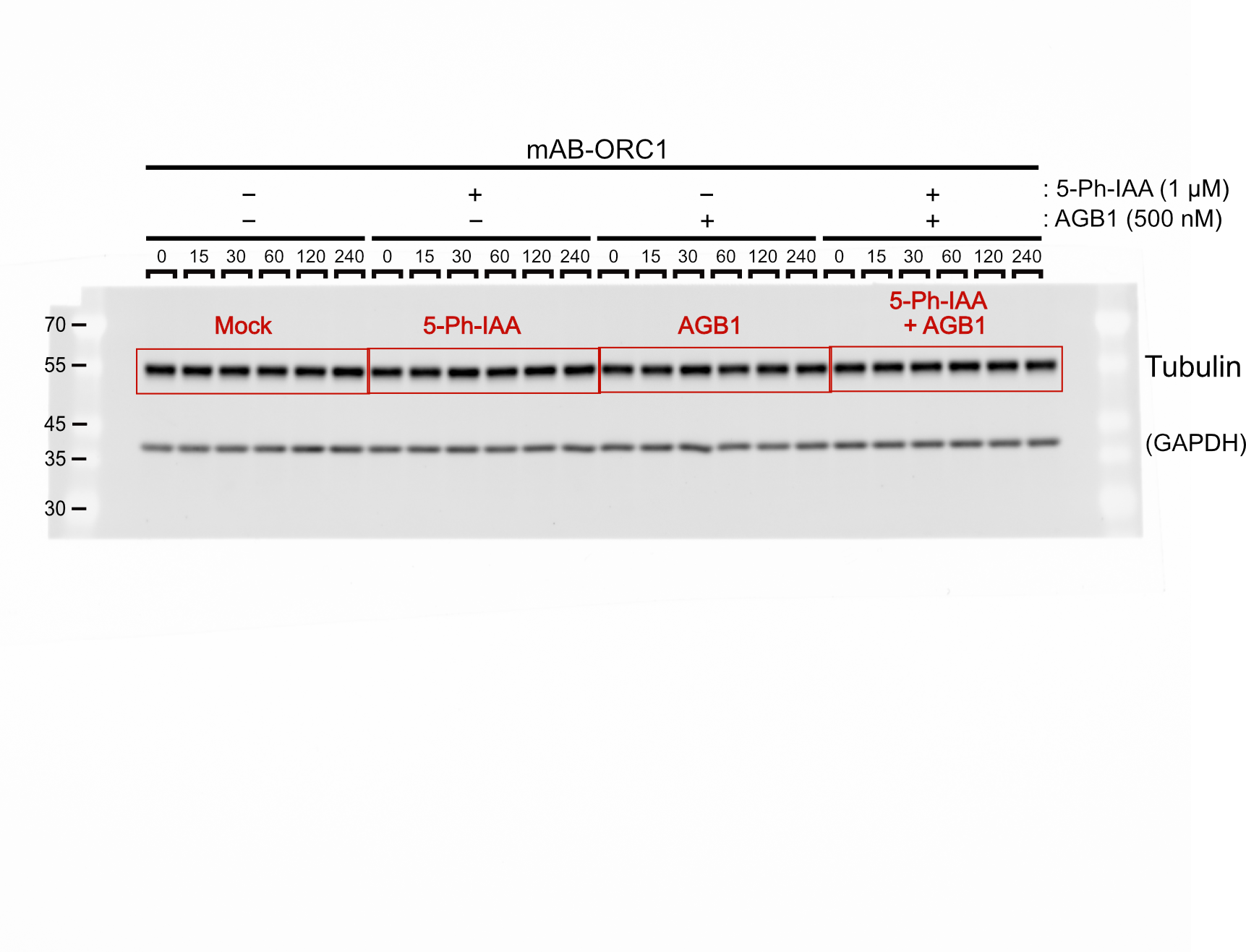

Supplement: Supplementary file 10 — Source data Fig. 5 [file 44319_2024_224_MOESM10_ESM.zip › Figure 5/5B/5B_Tubulin_WB.tiff]

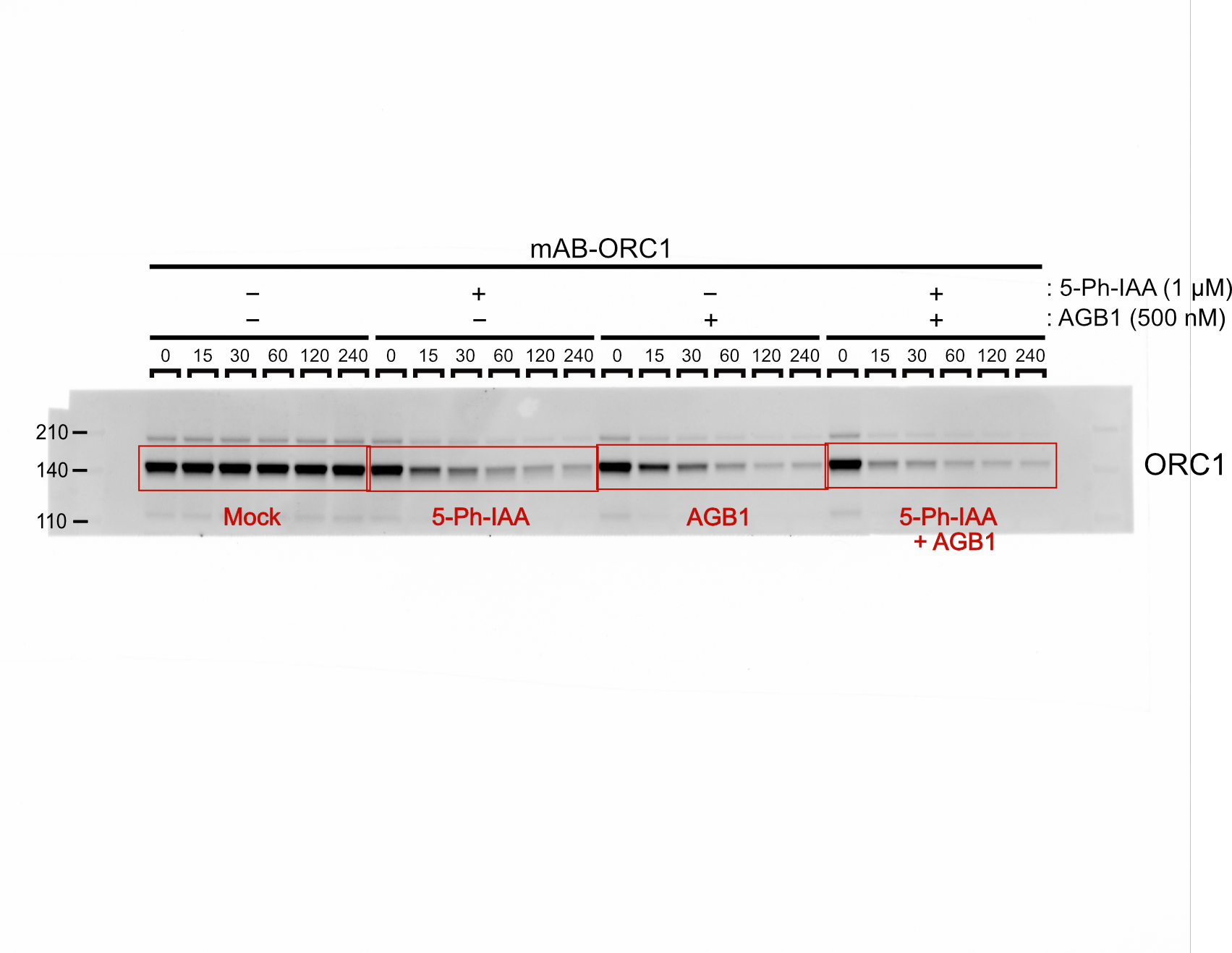

Supplement: Supplementary file 10 — Source data Fig. 5 [file 44319_2024_224_MOESM10_ESM.zip › Figure 5/5B/5B_ORC1_WB.tiff]

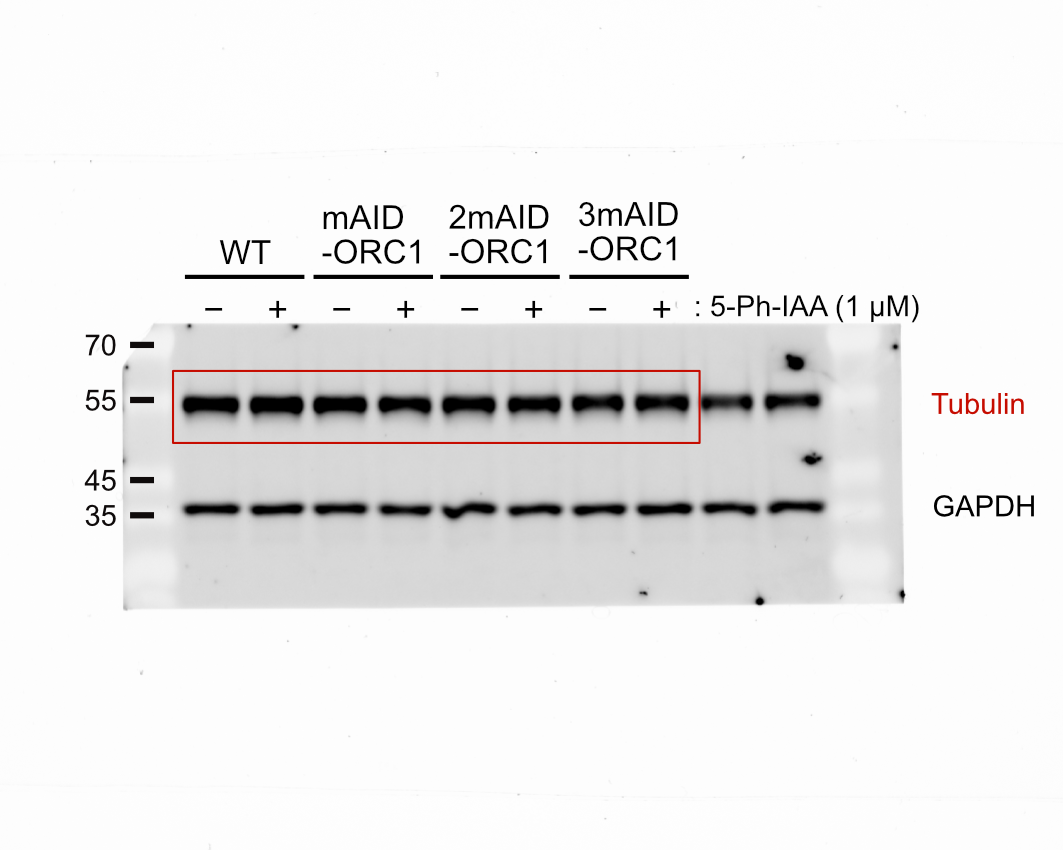

Supplement: Supplementary file 11 — Source data Fig. 6 [file 44319_2024_224_MOESM11_ESM.zip › Figure 6/6A/6A_Tubulin_WB.tiff]

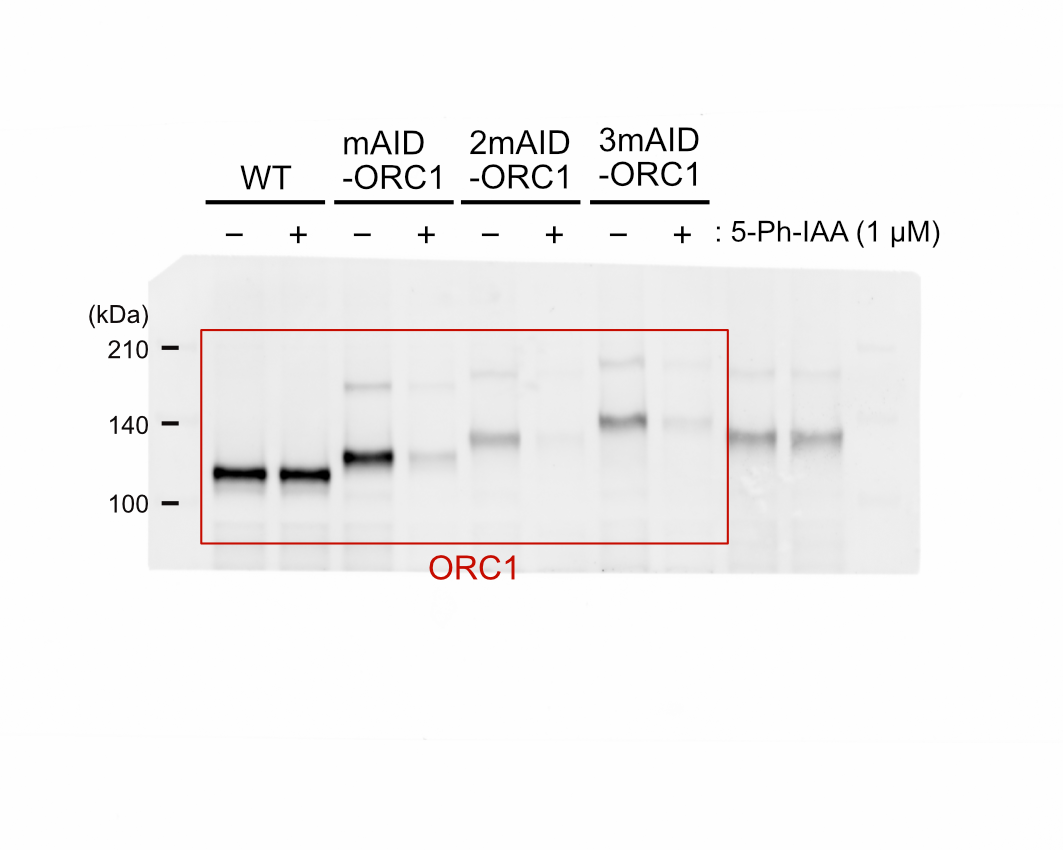

Supplement: Supplementary file 11 — Source data Fig. 6 [file 44319_2024_224_MOESM11_ESM.zip › Figure 6/6A/6A_ORC1_WB.tiff]

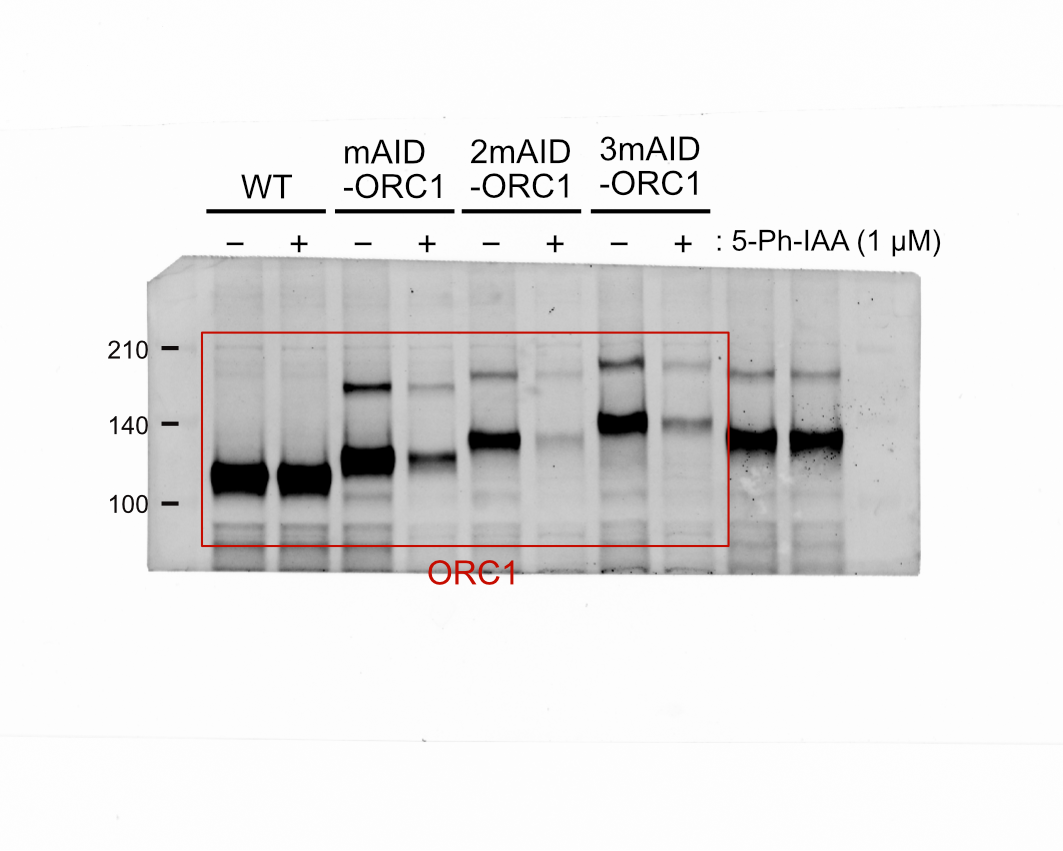

Supplement: Supplementary file 11 — Source data Fig. 6 [file 44319_2024_224_MOESM11_ESM.zip › Figure 6/6A/6A_ORC1_long exp_WB.tiff]

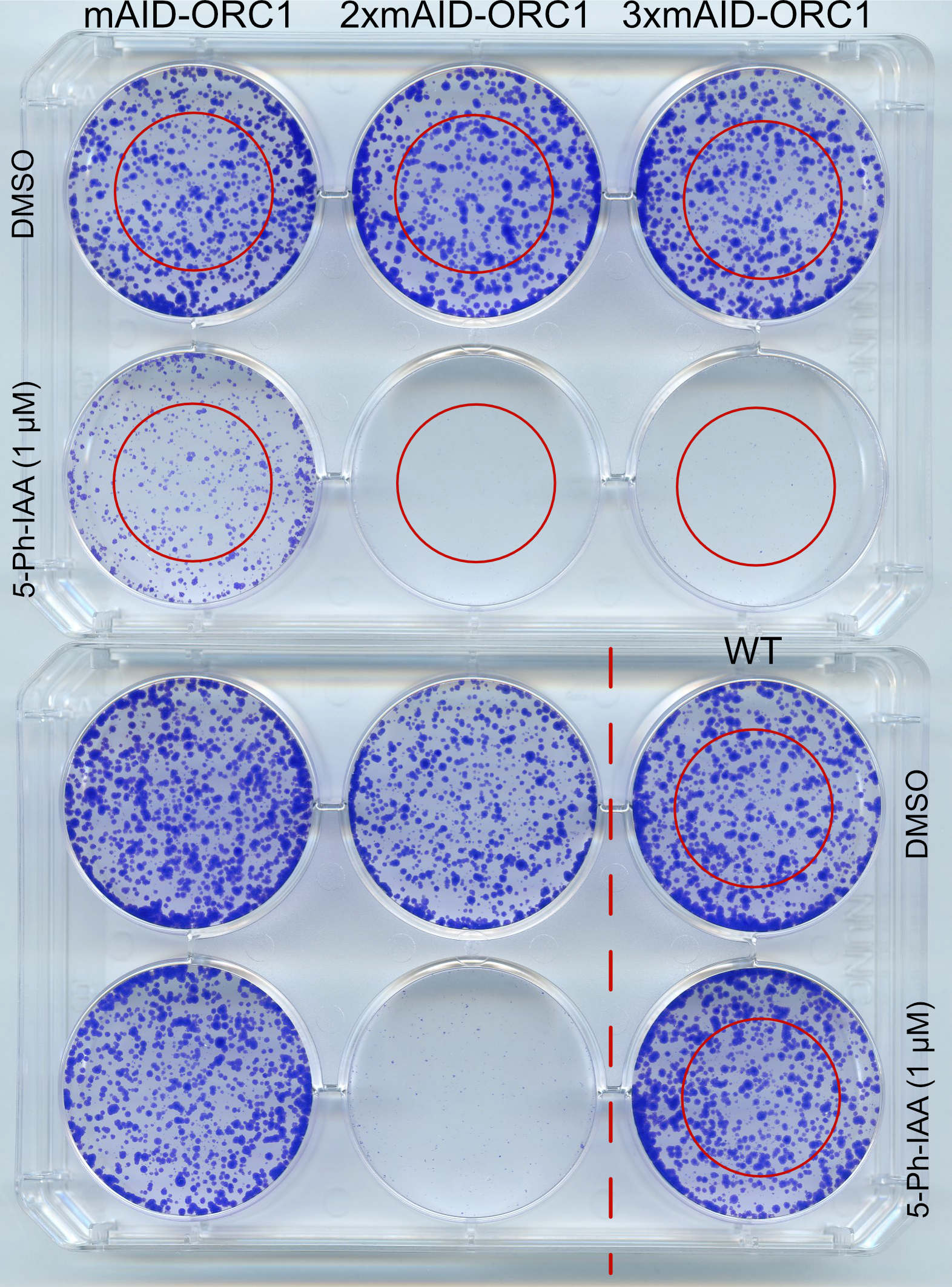

Supplement: Supplementary file 11 — Source data Fig. 6 [file 44319_2024_224_MOESM11_ESM.zip › Figure 6/6B/6B_Colony formation assay.tiff]

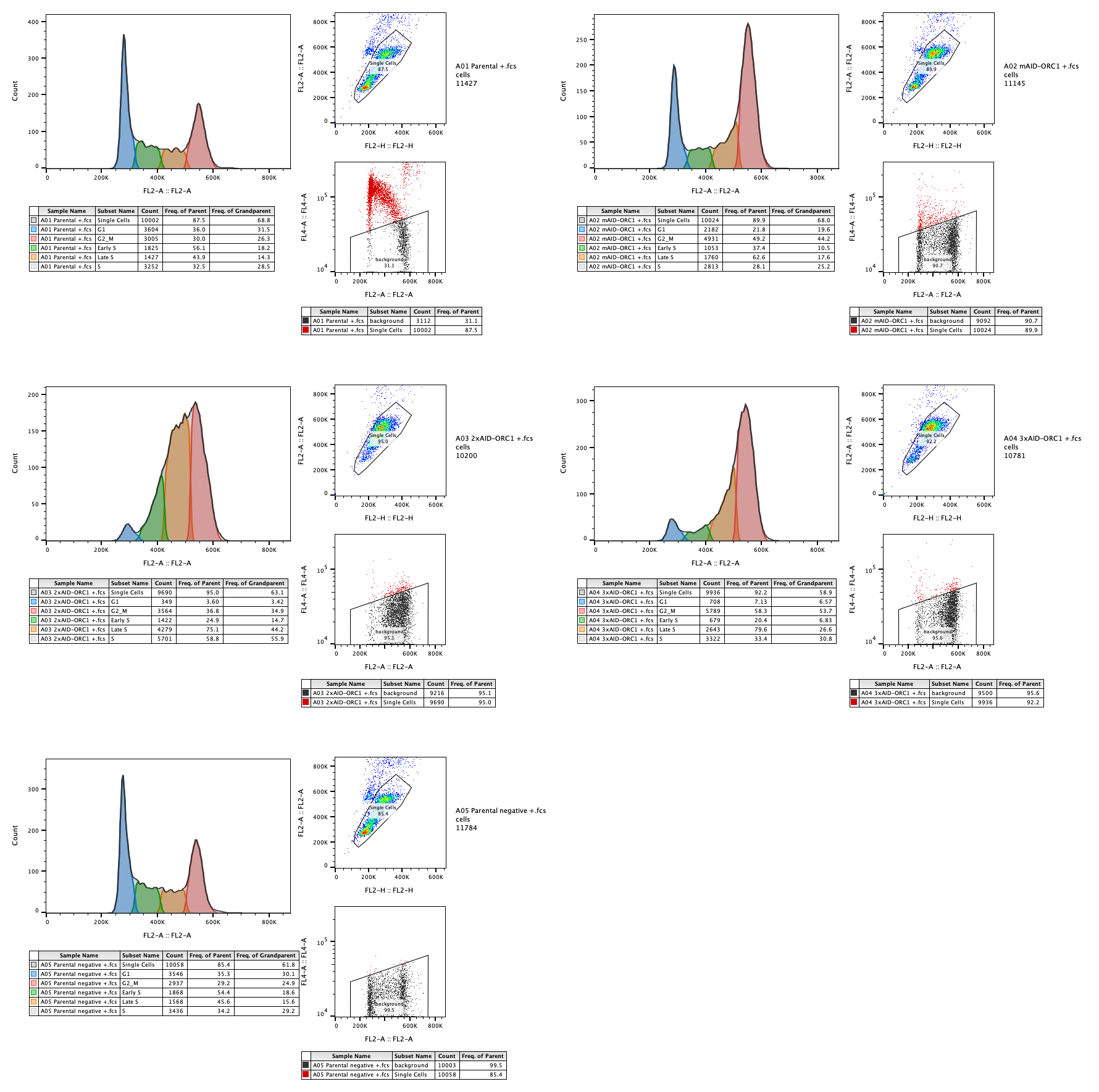

Supplement: Supplementary file 11 — Source data Fig. 6 [file 44319_2024_224_MOESM11_ESM.zip › Figure 6/6C/6C_Flowcyto.tiff]

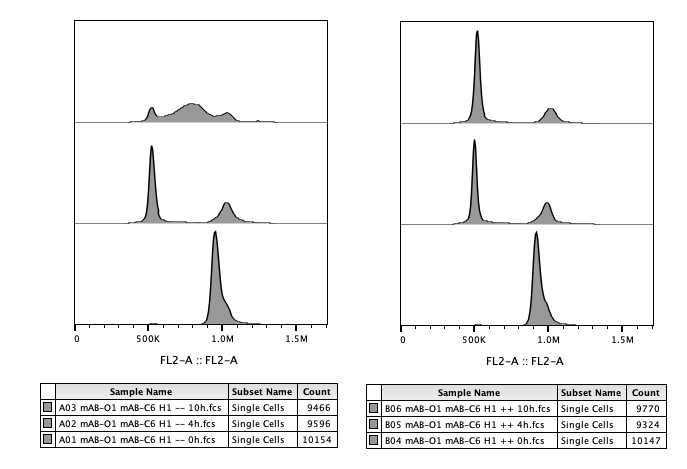

Supplement: Supplementary file 12 — Source data Fig. 7 [file 44319_2024_224_MOESM12_ESM.zip › Figure 7/7B/7B_Flowcyto.tiff]

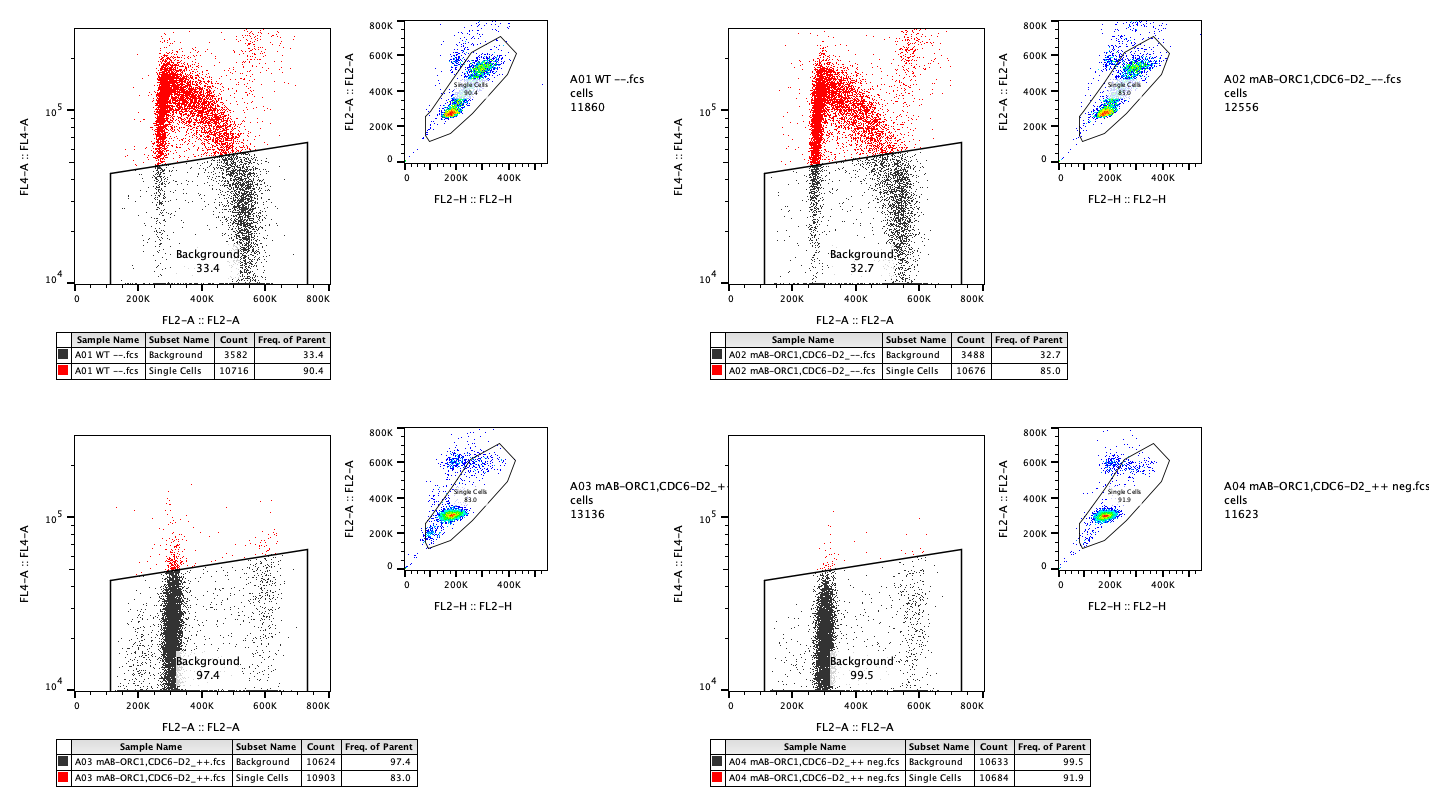

Supplement: Supplementary file 12 — Source data Fig. 7 [file 44319_2024_224_MOESM12_ESM.zip › Figure 7/7A/7A_flowcyto.tiff]

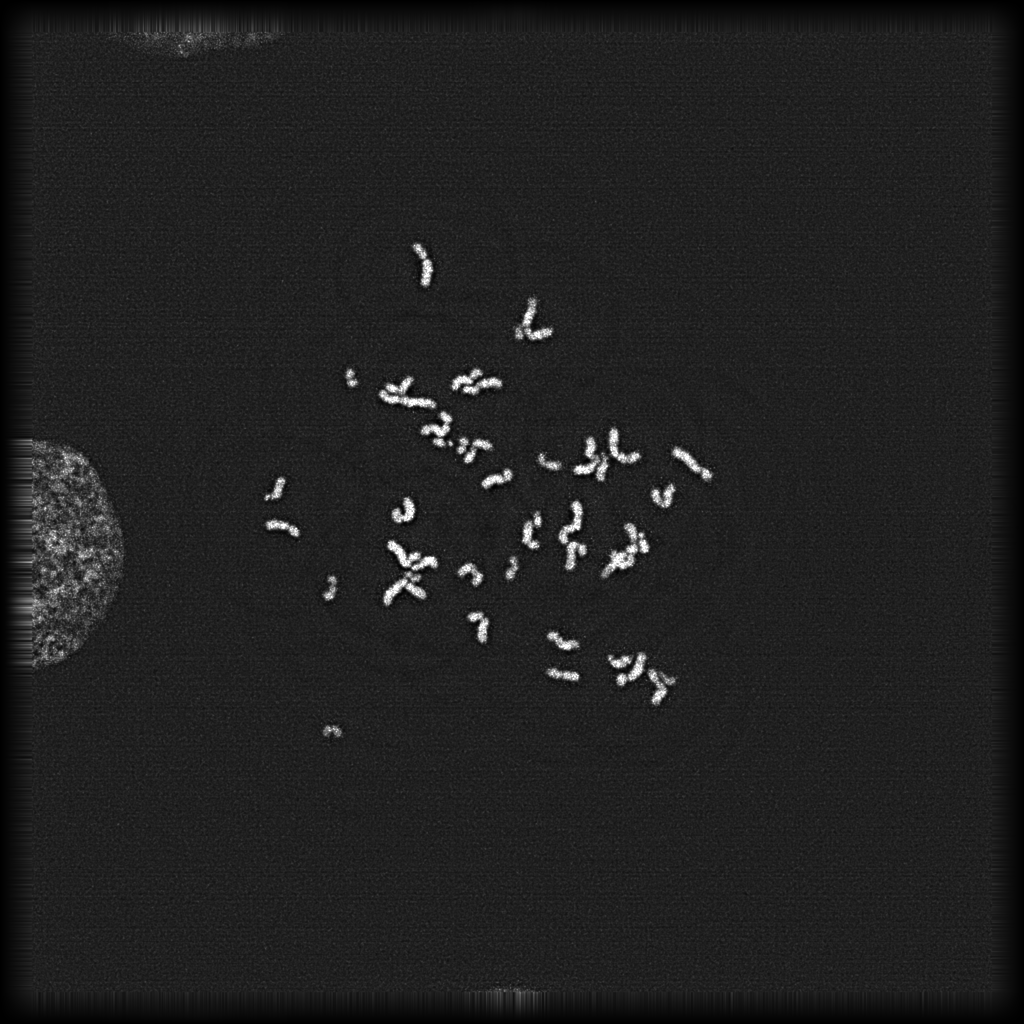

Supplement: Supplementary file 12 — Source data Fig. 7 [file 44319_2024_224_MOESM12_ESM.zip › Figure 7/7D/7D decon/mAB-ORC1 mAB-CDC6_5-Ph-IAA+AGB1_decon.tif]

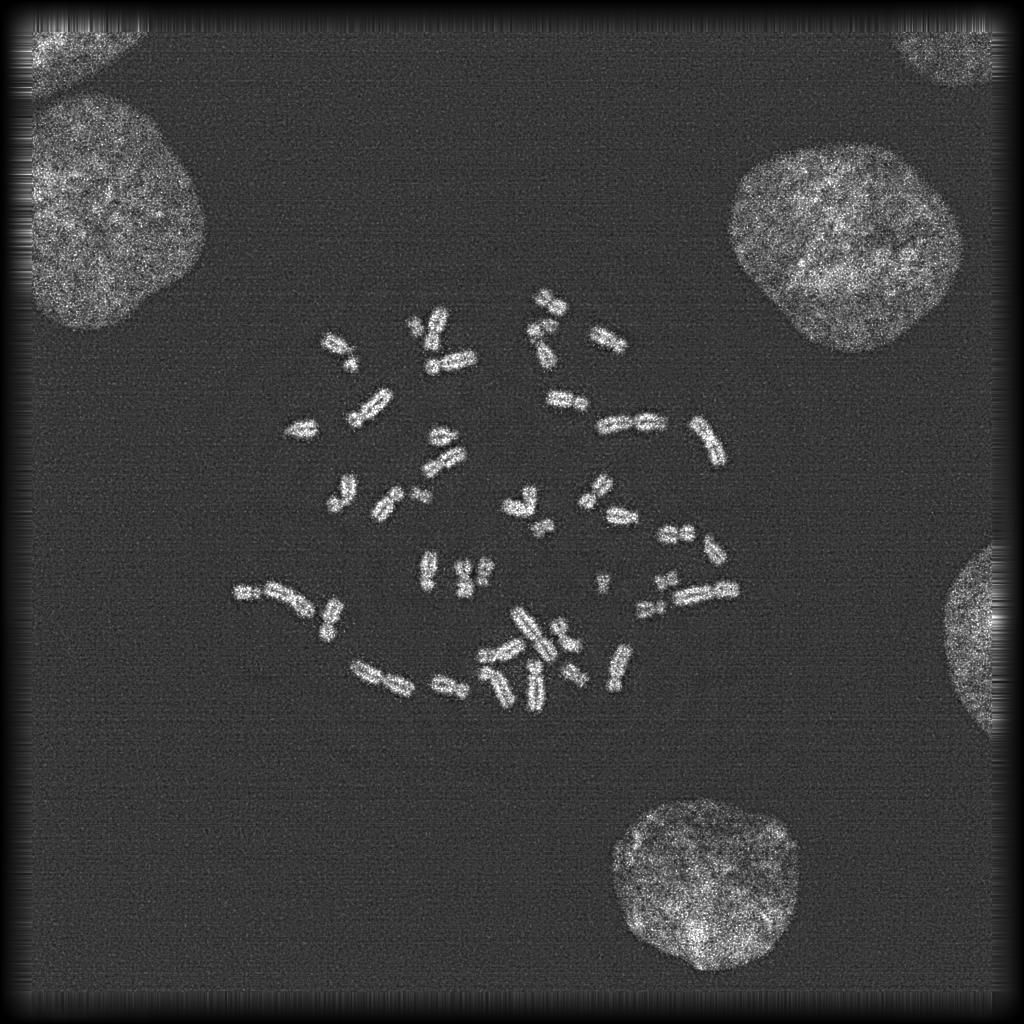

Supplement: Supplementary file 12 — Source data Fig. 7 [file 44319_2024_224_MOESM12_ESM.zip › Figure 7/7D/7D decon/mAB-ORC1 mAB-CDC6_DMSO_decon.tif]

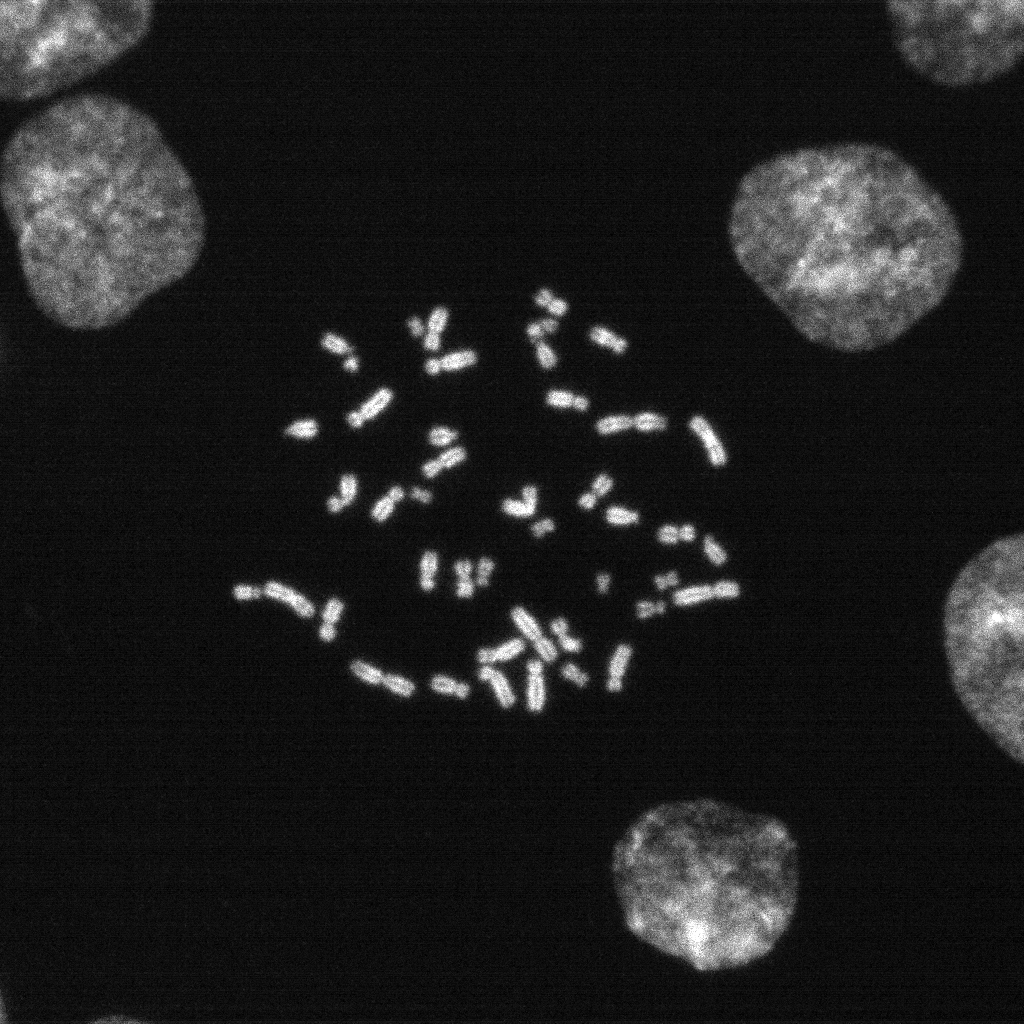

Supplement: Supplementary file 12 — Source data Fig. 7 [file 44319_2024_224_MOESM12_ESM.zip › Figure 7/7D/7D raw/mAB-ORC1 mAB-CDC6_DMSO.tif]

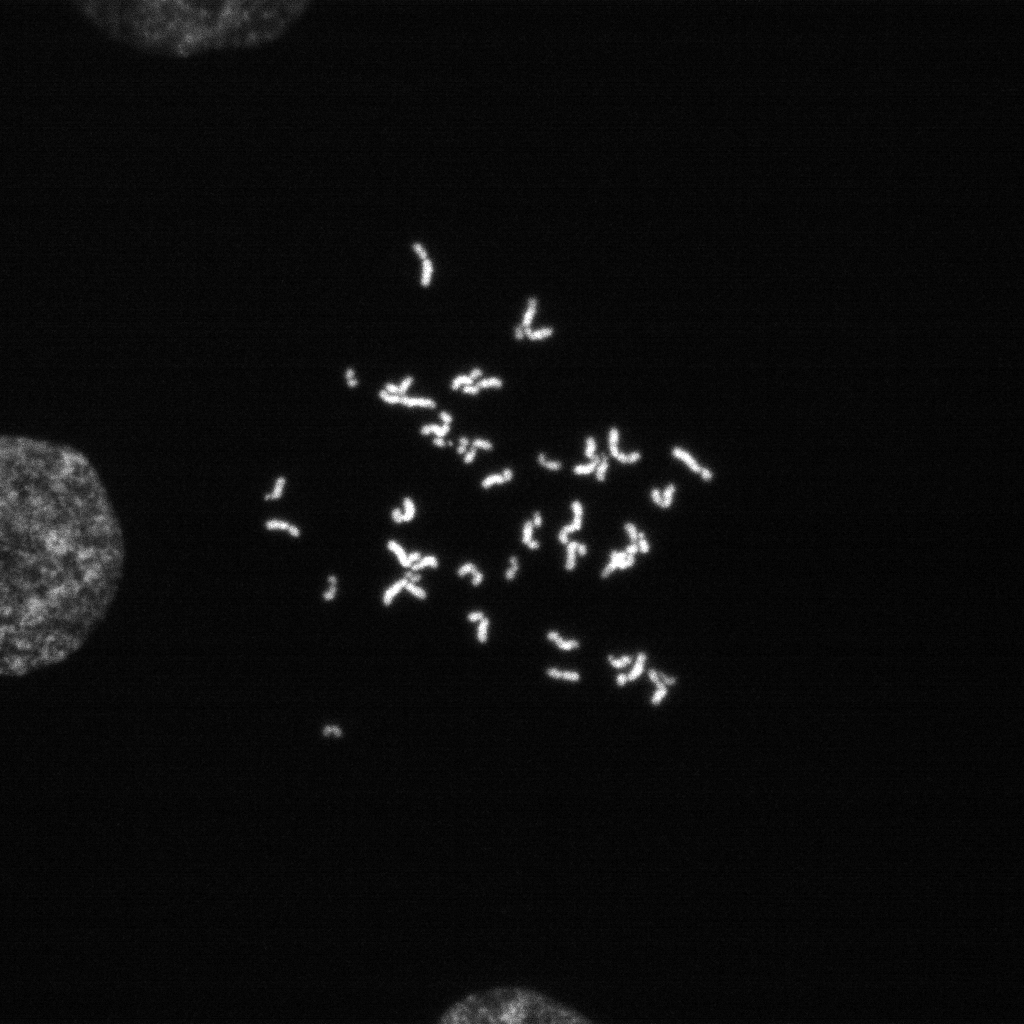

Supplement: Supplementary file 12 — Source data Fig. 7 [file 44319_2024_224_MOESM12_ESM.zip › Figure 7/7D/7D raw/mAB-ORC1 mAB-CDC6_5-Ph-IAA+AGB1.tif]

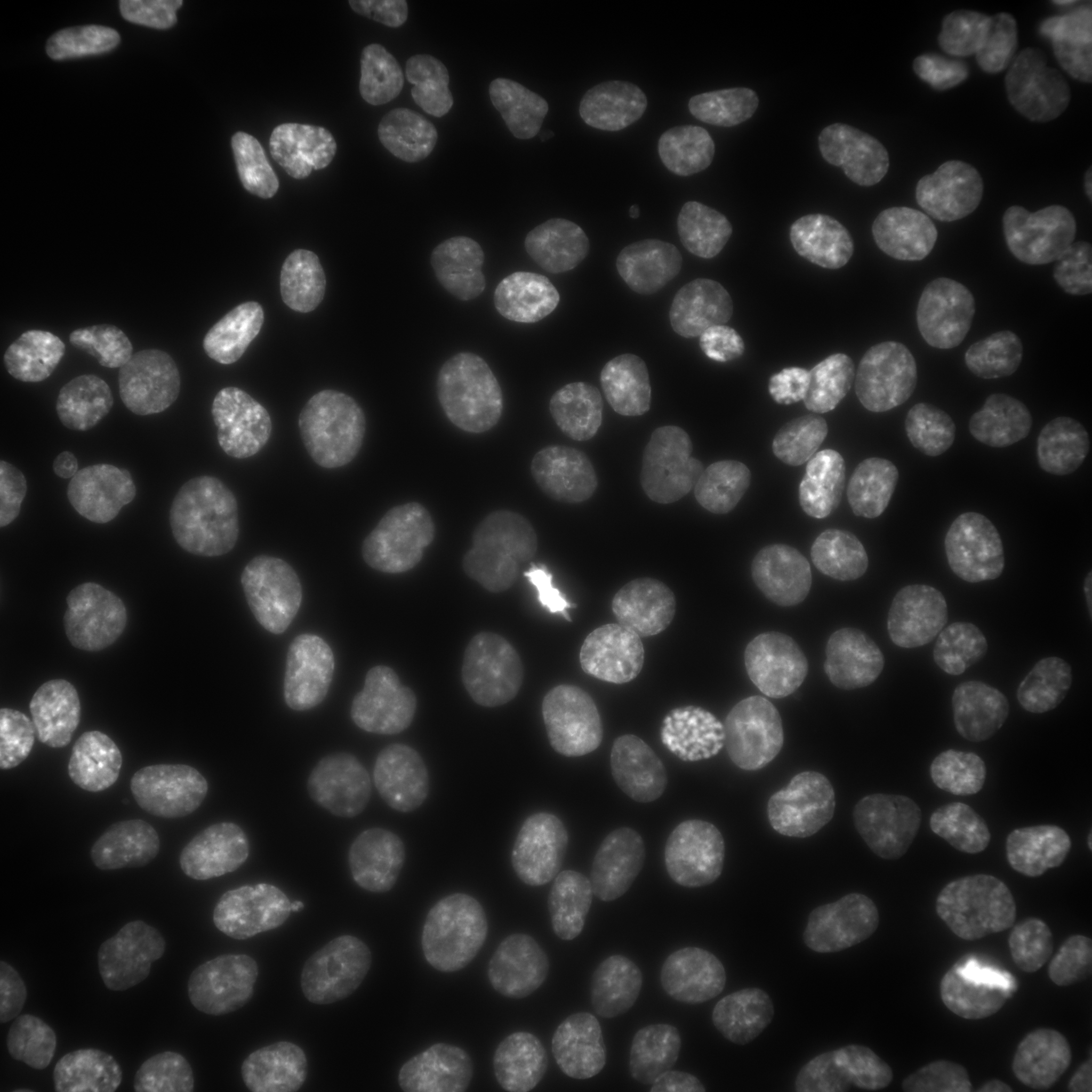

Supplement: Supplementary file 12 — Source data Fig. 7 [file 44319_2024_224_MOESM12_ESM.zip › Figure 7/7C/7C_raw/mAB-ORC1 mAB-CDC6_DMSO_DAPI_H3pS10_EdU.tif]

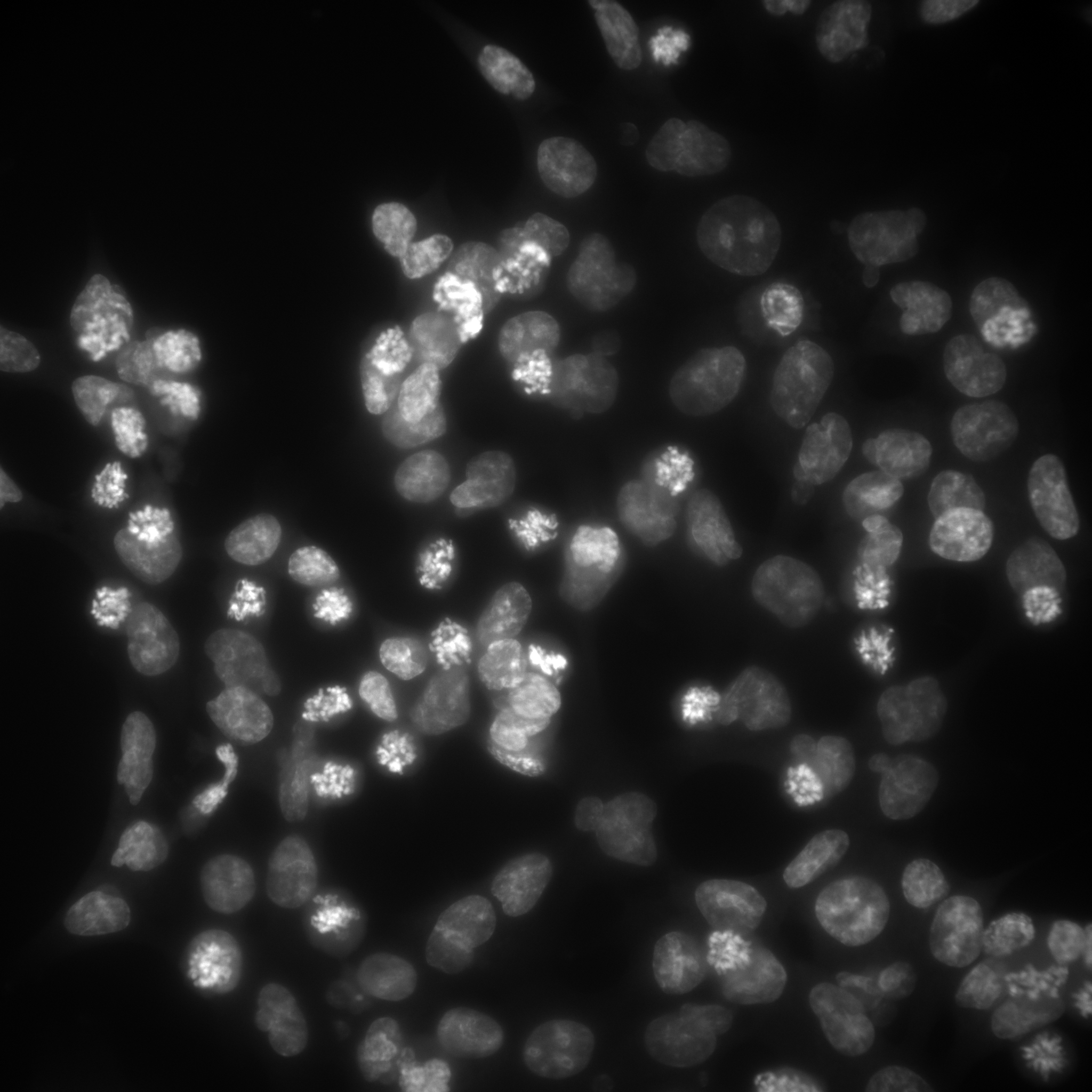

Supplement: Supplementary file 12 — Source data Fig. 7 [file 44319_2024_224_MOESM12_ESM.zip › Figure 7/7C/7C_raw/mAB-ORC1 mAB-CDC6_5Ph-IAA+AGB1_DAPI_H3pS10_EdU.tif]

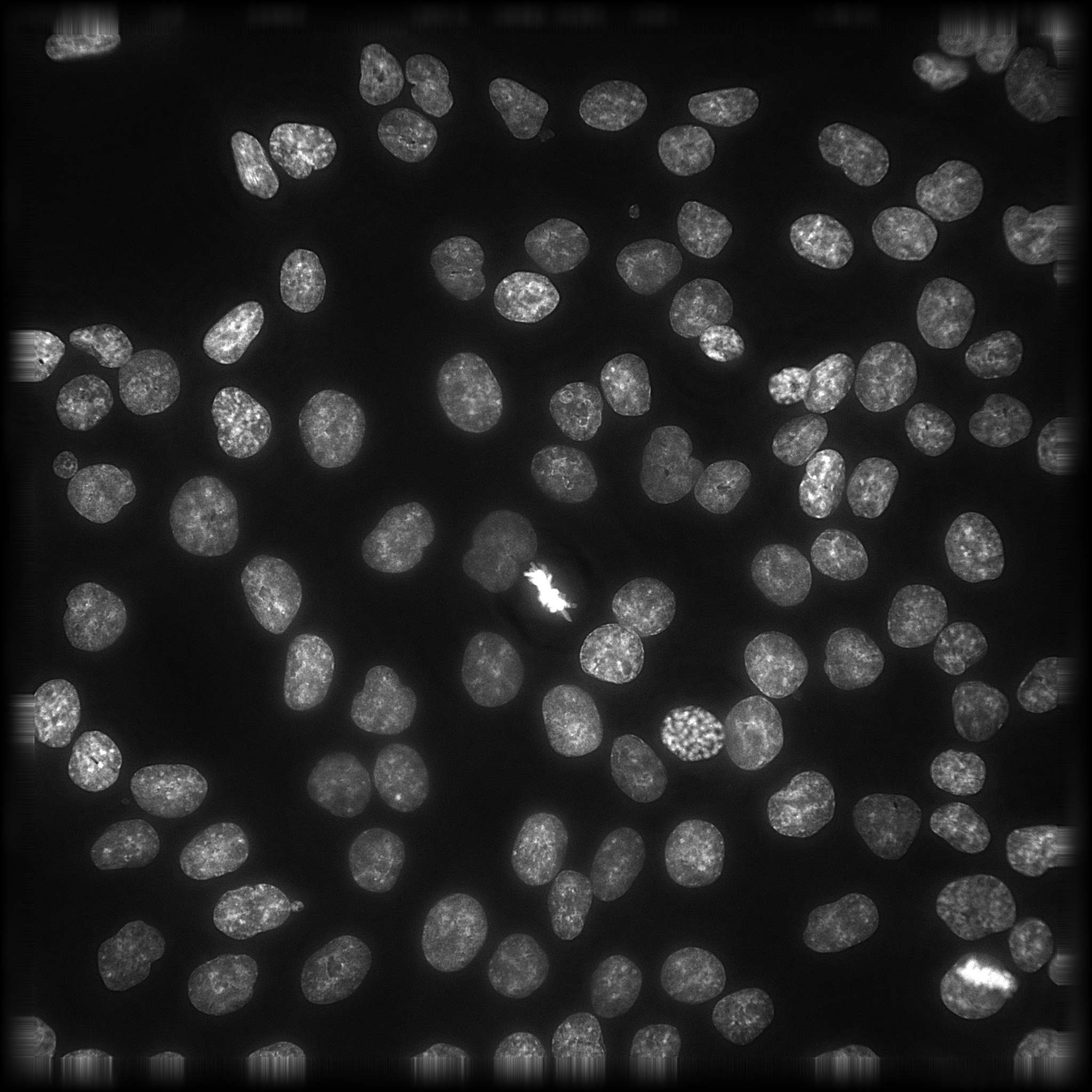

Supplement: Supplementary file 12 — Source data Fig. 7 [file 44319_2024_224_MOESM12_ESM.zip › Figure 7/7C/7C_deconvoluted/mAB-ORC1 mAB-CDC6_DMSO_DAPI_H3pS10_EdU_decon.tif]

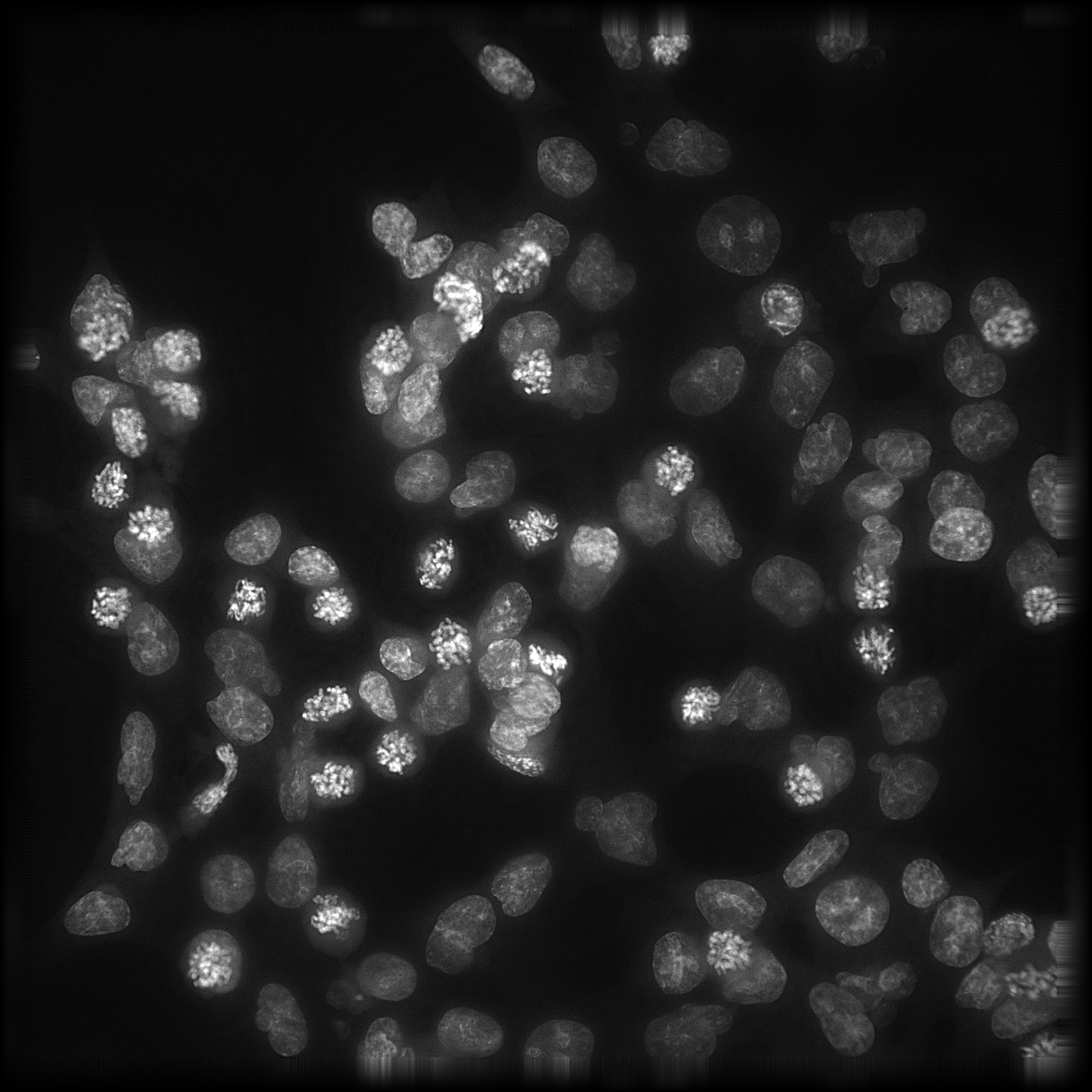

Supplement: Supplementary file 12 — Source data Fig. 7 [file 44319_2024_224_MOESM12_ESM.zip › Figure 7/7C/7C_deconvoluted/mAB-ORC1 mAB-CDC6_5Ph-IAA+AGB1_DAPI_H3pS10_EdU_decon.tif]
